# PD-1 instructs a tumor-suppressive metabolic program that restricts glycolysis and restrains AP-1 activity in T cell lymphoma

**DOI:** 10.1038/s43018-023-00635-7

**Published:** 2023-09-18

**Authors:** Tim Wartewig, Jay Daniels, Miriam Schulz, Erik Hameister, Abhinav Joshi, Joonhee Park, Emma Morrish, Anuroop V. Venkatasubramani, Filippo M. Cernilogar, Frits H. A. van Heijster, Christian Hundshammer, Heike Schneider, Filippos Konstantinidis, Judith V. Gabler, Christine Klement, Henry Kurniawan, Calvin Law, Yujin Lee, Sara Choi, Joan Guitart, Ignasi Forne, Jérôme Giustinani, Markus Müschen, Salvia Jain, David M. Weinstock, Roland Rad, Nicolas Ortonne, Franz Schilling, Gunnar Schotta, Axel Imhof, Dirk Brenner, Jaehyuk Choi, Jürgen Ruland

**Affiliations:** 1 TranslaTUM, Center for Translational Cancer Research, Technical University of Munich, Munich, Germany; 2 Institute of Clinical Chemistry and Pathobiochemistry, School of Medicine, Technical University of Munich, Munich, Germany; 3 Center of Molecular and Cellular Oncology, Yale School of Medicine, Yale University, New Haven, CT USA; 4 Department of Biochemistry and Molecular Genetics, Northwestern University, Feinberg School of Medicine, Chicago, IL USA; 5 Department of Dermatology, Northwestern University, Feinberg School of Medicine, Chicago, IL USA; 6 German Cancer Consortium (DKTK), Heidelberg, Germany; 7 Protein Analysis Unit, Biomedical Center, Faculty of Medicine, Ludwig-Maximilians-Universität Munich, Martinsried, Germany; 8 Department of Molecular Biology, Biomedical Center, Faculty of Medicine, Ludwig-Maximilians-Universität Munich, Martinsried, Germany; 9 Department of Nuclear Medicine, School of Medicine, Technical University of Munich, Munich, Germany; 10 Institute of Molecular Oncology and Functional Genomics, School of Medicine, Technical University of Munich, Munich, Germany; 11 Experimental and Molecular Immunology, Department of Infection and Immunity, Luxembourg Institute of Health, Esch-sur-Alzette, Luxembourg; 12 Immunology and Genetics, Luxembourg Centre for Systems Biomedicine, University of Luxembourg, Belvaux, Luxembourg; 13 Institut Mondor de Recherche Biomédicale, Inserm U955, Paris-Est Créteil University, Créteil, France; 14 Department of Immunobiology, Yale University, New Haven, CT USA; 15 Dana–Farber Cancer Institute, Harvard Medical School, Boston, MA USA; 16 Department of Medicine II, School of Medicine, Technical University of Munich, Munich, Germany; 17 Pathology Department, AP-HP Inserm U955, Henri Mondor Hospital, Créteil, France; 18 Odense Research Center for Anaphylaxis, Department of Dermatology and Allergy Center, Odense University Hospital, University of Southern Denmark, Odense, Denmark; 19 Robert H. Lurie Comprehensive Cancer Center, Northwestern University, Chicago, IL USA; 20 Center for Genetic Medicine, Northwestern University, Feinberg School of Medicine, Chicago, IL USA; 21 Center for Human Immunobiology, Northwestern University, Feinberg School of Medicine, Chicago, IL USA; 22 Center for Synthetic Biology, Northwestern University, Evanston, IL USA; 23 German Center for Infection Research (DZIF), partner site Munich, Munich, Germany; 24 Present Address: Merck Research Laboratories, Boston, MA USA

**Keywords:** Haematological cancer, Signal transduction, Cancer, Cancer metabolism

## Abstract

The *PDCD1*-encoded immune checkpoint receptor PD-1 is a key tumor suppressor in T cells that is recurrently inactivated in T cell non-Hodgkin lymphomas (T-NHLs). The highest frequencies of *PDCD1* deletions are detected in advanced disease, predicting inferior prognosis. However, the tumor-suppressive mechanisms of PD-1 signaling remain unknown. Here, using tractable mouse models for T-NHL and primary patient samples, we demonstrate that PD-1 signaling suppresses T cell malignancy by restricting glycolytic energy and acetyl coenzyme A (CoA) production. In addition, PD-1 inactivation enforces ATP citrate lyase (ACLY) activity, which generates extramitochondrial acetyl-CoA for histone acetylation to enable hyperactivity of activating protein 1 (AP-1) transcription factors. Conversely, pharmacological ACLY inhibition impedes aberrant AP-1 signaling in PD-1-deficient T-NHLs and is toxic to these cancers. Our data uncover genotype-specific vulnerabilities in *PDCD1*-mutated T-NHL and identify PD-1 as regulator of AP-1 activity.

## Abstract (web summary)

Wartewig et al. show that *PDCD1* in T cell lymphoma restricts glycolytic energy and acetyl-CoA production. Inhibition of PD-1 signaling enforces ACLY activity and generates extramitochondrial acetyl-CoA for histone acetylation to enable AP-1 hyperactivation.

## Main

T cell non-Hodgkin lymphomas (T-NHLs) represent a heterogeneous group of highly aggressive cancers that typically originate from mature CD4^+^ T cells^1^. The therapeutic options for these malignancies are limited, which is largely due to their ill-defined molecular pathogenesis^1^. However, recent genomic analyses of large cohorts of patients with T-NHL revealed numerous oncogenic, gain-of-function alterations in T cell antigen receptor (TCR) signaling pathways. In addition, *PDCD1*, which encodes the inhibitory immune receptor PD-1, emerged as a key tumor suppressor in T-NHL^2^. Inactivating mutations in *PDCD1* predict an aggressive clinical phenotype, and they portend poor overall survival in patients^2^. In addition, anti-PD-1 checkpoint inhibitors have been correlated with the emergence of secondary T-NHLs in patients with other primary malignancies^3,4^. Moreover, clinical trials in patients with T-NHL have reported hyperprogression of individual T cell lymphomas, which were apparently still under PD-1 control, after anti-PD-1 treatment (NCT02631746 (refs. ^5,6^), NCT03075553 (ref. ^7^)). While all these clinical observations highlight the critical importance of inhibitory PD-1 signaling in T cell malignancies, the tumor-suppressive mechanisms of PD-1 remain unknown.

In non-transformed T cells, acute PD-1 engagement leads to inhibition of the (phosphoinositide 3-kinase (PI3K))–AKT axis^8^, and persistent PD-1 signaling triggers T cell exhaustion, a dysfunctional state with specific metabolic and epigenetic characteristics^9,10^. In addition, RNA-sequencing (RNA-seq) analysis from primary human and murine PD-1-deficient T-NHLs^2^ demonstrates that these tumors are characterized by distinct gene expression that is presumably also shaped by epigenetic mechanisms. Nevertheless, the molecular link between PD-1 signaling and metabolic and epigenetic reprogramming during lymphomagenesis remains unclear.

Here, we identify PD-1 as a major gatekeeper for the oncogene-triggered metabolic switch to glycolysis. We show that *PDCD1* mutations enforce the induction of key metabolic molecules for glucose uptake, metabolization and generation of energy carriers. In addition, PD-1 regulates ACLY, which uses extramitochondrial citrate to fuel acetyl-CoA pools for histone acetylation and enables aberrant AP-1 activity in the tumor cells. These AP-1-inducing mechanisms are hijacked in aggressive PD-1-deficient lymphomas but not in their PD-1-competent counterparts and link PD-1 signaling to epigenetic reprogramming in T-NHL.

## Results

**Fig. 1:**
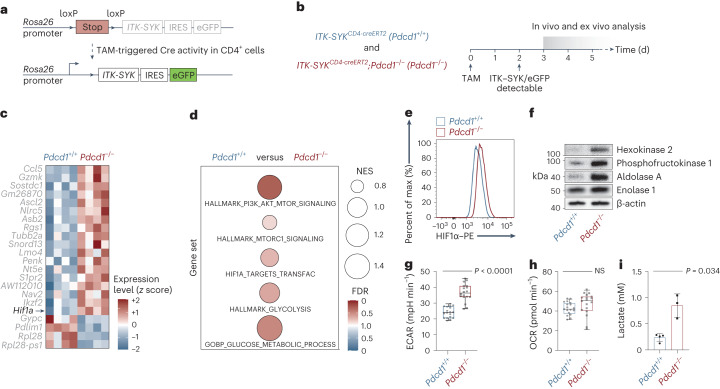
Loss of *Pdcd1* enables oncogene-enforced glycolysis in T cells. **a**, Transgenic *ITK*-*SYK* allele with eGFP reporter sequence and Cre-induced excision of the stop cassette. IRES, internal ribosome entry site; TAM, tamoxifen. **b**, Experimental strategy to explore early molecular events upon ITK–SYK induction in the presence or absence of *Pdcd1*. **c**, Top 20 differentially expressed genes between *ITK-SYK*^CD4-CreERT2^ (‘*Pdcd1*^+/+^’)- and *ITK-SYK*^CD4-CreERT2^;*Pdcd1*^−/−^ (‘*Pdcd1*^−/−^’)-derived ITK–SYK-expressing T cells. RNA-seq was performed using spleen-derived eGFP^+^ T cells, sorted by flow cytometry on day 5 after tamoxifen injection (*n* = 4 mice per group). Expression values are normalized *z* scores. **d**, GSEA of the indicated signatures. FDR, color intensity of circles; NES, circle diameter. Blue and red indicate the group in which the signature was positively enriched; NES, normalized enrichment score; FDR, false discovery rate. **e**, Flow cytometry for HIF1α in ITK–SYK-expressing T cells. Spleen-derived single-cell suspensions were generated from *ITK-SYK*^CD4-CreERT2^ and *ITK-SYK*^CD4-CreERT2^;*Pdcd1*^−/−^ mice on day 5 after tamoxifen injection. Max, maximum. **f**, Western blot analysis from lysates of ITK–SYK-expressing eGFP^+^ T cells, sorted by flow cytometry from *ITK-SYK*^CD4-CreERT2^ and *ITK-SYK*^CD4-CreERT2^;*Pdcd1*^−/−^ mice on day 5 after tamoxifen injection. **g**, ECAR metabolic flux analysis of ITK–SYK-expressing CD4^+^ T cells isolated from *ITK-SYK*^CD4-CreERT2^ and *ITK-SYK*^CD4-CreERT2^;*Pdcd1*^−/−^ mice on day 5 after tamoxifen injection (*n* = 3 biological replicates per group). Data were normalized using total cellular protein. *P*, two-sided Student’s *t*-test. Middle line denotes the median, the top and bottom box edges denote 0.25 and 0.75 quantiles, respectively, and the whiskers denote the minimum and maximum values. **h**, OCR metabolic flux analysis from the same experiment as in **g**. *P*, two-sided Student’s *t*-test. **i**, Lactate concentration in cell culture supernatants. ITK–SYK-expressing eGFP^+^ cells were sorted by flow cytometry from *ITK-SYK*^CD4-CreERT2^ and *ITK-SYK*^CD4-CreERT2^;*Pdcd1*^−/−^ mice on day 5 after tamoxifen injection and incubated overnight in vitro (*n* = 3 and *n* = 4 biological replicates per group). Data were normalized using viable cell numbers. *P*, two-sided Student’s *t*-test. Shown are the mean ± s.d. and individual data points. **c**,**d**, Data from one experiment. **e**,**f**, Representative data from two independent experiments with two biological replicates per group. **g**,**h**, Representative data from two independent experiments with three biological replicates per group. **i**, Representative data from two independent experiments. Source data

**Extended Data Fig. 1:**
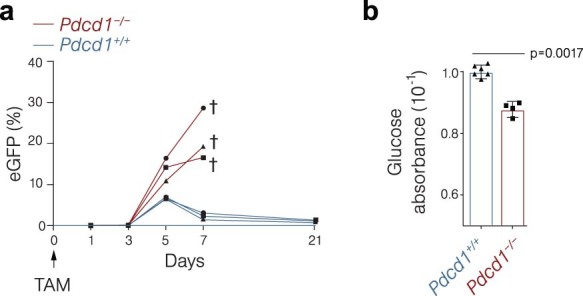
Increased glucose uptake upon *Pdcd1* loss. **a**, Percentage of eGFP-expressing cells as determined by flow cytometric analysis of peripheral blood. A cross indicates death of the animal. Individual data points are shown (n = 3 mice per genotype). TAM, tamoxifen. **b**, Remaining glucose in cell culture supernatants. ITK-SYK-expressing T cells were sorted by FACS for eGFP^+^ from the spleens of acutely induced *ITK-SYK*^*CD4-creERT2*^ (‘*Pdcd1*^*+/+*^*’*) and *ITK-SYK*^*CD4-creERT2*^*;Pdcd1*^−/−^ mice (‘*Pdcd1*^−/−^*‘*) and cultured *in vitro* for four hours. Next, the cell culture supernatants were harvested, and glucose concentrations were determined by colorimetric measurements. All animals received single-dose intraperitoneal injections of tamoxifen five days before the experiment (n = 6 and n = 4 mice per genotype). Data was normalized to viable cell number. P=two-sided Student’s t-test. Shown are the mean ± SD and individual data points. **a**, Representative results from 8 independent experiments with three biological replicates per group. **b**, Representative results from 2 independent experiments. Source data

To dissect T cell lymphoma pathogenesis, we previously engineered a genetically controllable murine model of human T-NHL based on tamoxifen-inducible irreversible expression of the T-NHL oncogene *ITK*-*SYK*, which enforces strong oncogenic TCR signaling^11^, together with an enhanced green fluorescent protein (eGFP) reporter in individual mature CD4^+^ T cells in vivo (*ITK*-*SYK*^CD4-CreERT2^ mice; for the experimental system, see Fig. 1a,b)^11,12^. While acute expression of interleukin (IL)-2-inducible T cell kinase (ITK)–spleen-associated tyrosine kinase (SYK) (together with eGFP) in otherwise unperturbed primary T cells can trigger lymphocyte proliferation for a few days, it is unable to induce overt malignancy, which requires the acquisition of additional genetic hits (Extended Data Fig. 1a)^11,12^. However, loss of the tumor-suppressor gene *Pdcd1* is sufficient to enable immediate unrestricted clonal expansion of lymphomatous T cells upon single oncogene expression that is lethal to the host (Extended Data Fig. 1a)^12^. These cells can transmit the malignant disease to secondary recipients^12^. We leveraged this genetically tractable mouse model for human T-NHL to investigate the very early events of T cell transformation.

### Loss of *Pdcd1* enables oncogene-enforced glycolysis

To identify the potent PD-1-controlled tumor-suppressor programs that prevent T cell transformation upon oncogenic T cell signaling, we induced ITK–SYK expression in vivo in primary CD4^+^ T cells with and without *Pdcd1* by injecting tamoxifen into *ITK-SYK*^CD4-CreERT2^ and *ITK-SYK*^CD4-CreERT2^;*Pdcd1*^−/−^ mice. Three days after ITK–SYK expression, we purified oncogene-expressing cells by flow cytometry for RNA-seq. Among the top 20 differentially expressed genes, *Hif1a*, encoding a master regulator of cellular metabolism, was specifically upregulated in the absence of *Pdcd1* (Fig. 1c; adjusted *P* < 0.0001). In addition, gene set enrichment analysis (GSEA) revealed an enrichment of multiple gene expression signatures that indicate global activation of the PI3K–AKT–mammalian target of rapamycin (mTOR)–hypoxia-inducible factor (HIF)1α axis^13^ upon PD-1 loss. Furthermore, enrichment of multiple gene expression signatures suggests enhanced glucose metabolism in the PD-1-deficient lymphoma cells compared to their premalignant ITK–SYK^+^PD-1^+^ counterparts (Fig. 1d). These gene expression data were corroborated by protein expression analysis of HIF1α and several enzymes that mediate glycolysis, including hexokinase 2, phosphofructokinase 1, aldolase A and enolase 1 in ex vivo isolated, acute oncogene-expressing T cells from tamoxifen-injected *ITK-SYK*^CD4-CreERT2^ and *ITK-SYK*^CD4-CreERT2^;*Pdcd1*^−/−^ mice (Fig. 1e,f)^14,15^.

**Fig. 2:**
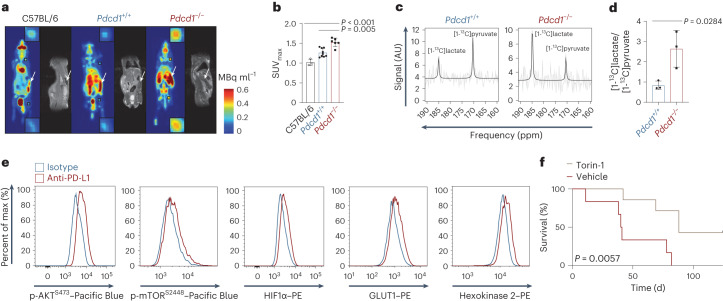
PD-1 represses mTOR and HIF1α in premalignant cells. **a**, Whole-body standardized uptake value (SUV) maps from [^18^F]FDG PET measurements of C57BL/6 mice*, ITK-SYK*^CD4-CreERT2^ and *ITK-SYK*^CD4-CreERT2^;*Pdcd1*^−/−^ mice together with anatomical MRI acquisition. The [^18^F]FDG PET measurements were conducted on day 5 after tamoxifen application. Magnified [^18^F]FDG PET signals from lymph nodes are shown (insets). White arrows indicate spleens. Color intensity represents the calculated uptake of [^18^F]FDG in units of MBq ml^−1^. **b**, SUV_max_ for the indicated genotypes (*n* = 3 for C57BL/6, *n* = 9 for *Pdcd1*^+/+^ and *n* = 6 for *Pdcd1*^−/−^ mice) from the experiment shown in **a**. SUV_max_ denotes the maximum signal found in a single voxel across the entire tumor lesion. *P*, one-way ANOVA and two-sided Student’s *t*-test. Shown are the mean ± s.e.m. and individual data points. **c**, Exemplary 7-T MRI chemical shift imaging (CSI)-derived ^13^C magnetic resonance spectra showing [1-^13^C]pyruvate and [1-^13^C]lactate amplitudes in the splenic region of interest of *ITK-SYK*^CD4-CreERT2^ and *ITK-SYK*^CD4-CreERT2^;*Pdcd1*^−/−^ mice following injection of hyperpolarized [1-^13^C]pyruvate on day 5 after tamoxifen injection. AU, arbitrary units. **d**, Ratio of [1-^13^C]pyruvate and [1-^13^C]lactate amplitudes in spleens measured by 7-T MRI in *ITK-SYK*^CD4-CreERT2^ and *ITK-SYK*^CD4-CreERT2^;*Pdcd1*^−/−^ mice on day 5 after tamoxifen injection (*n* = 3 mice per genotype). *P*, two-sided Student’s *t*-test. Shown are the mean ± s.d. and individual data points. **e**, Flow cytometry for p-AKT^S473^, p-mTOR^S2448^, HIF1α, GLUT1 and hexokinase 2 in ITK–SYK-expressing T cells. Spleen-derived single-cell suspensions were generated from *ITK-SYK*^CD4-CreERT2^ mice on day 5 after tamoxifen injection. Before fixation, anti-PD-L1 or control antibodies were added for 4 h in vitro. **f**, Survival of ITK–SYK^+^PD-1^−^ lymphoma-bearing NSG mice treated with torin-1 or vehicle. On day 0, NSG mice received 1,000 ITK–SYK-expressing eGFP^+^ cells from *an ITK-SYK*^CD4-CreERT2^;*Pdcd1*^−/−^ mouse, sorted by flow cytometry on day 5 after tamoxifen injection. Mice received 6 mg per kg torin-1 or vehicle daily via intraperitoneal injection (*n* = 6 mice per group) from day 3 post tamoxifen on. *P*, two-sided log-rank test. **a**, Representative mice from two independent experiments. **b**, Pooled data from two independent experiments. **c**, Representative amplitudes from one experiment. **d**, Pooled data from two independent experiments. **e**, Representative data from two independent experiments with two biological replicates per group. **f**, Pooled data from two independent experiments. Source data

**Extended Data Fig. 2:**
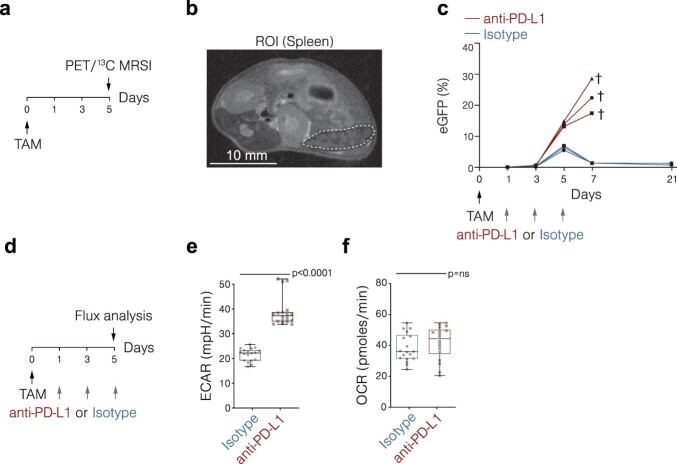
PD-1 inactivation enables oncogene-enforced glycolysis *in vivo*. **a**, Schematic outline of metabolic imaging analysis experiment. **b**, Exemplary splenic T1-weighted sequence of an acutely induced *ITK-SYK*^*CD4-creERT2*^*;Pdcd1*^−/−^ animals with indicated ROI used for 7T-MRI [1-^13^C]pyruvate and [1-^13^C]lactate measurements. ROI, region of interest. **c**, Percentage of viable lymphocytes in the peripheral blood of anti-PD-L1 or isotype control antibodies treated *ITK-SYK*^*CD4-creERT2*^ mice. All animals received a single dose of tamoxifen on day zero. Cross indicates death of the animal (n = 3 mice per genotype). **d**, Schematic outline of metabolic flux analysis experiment. **e**, ECAR metabolic flux analysis of ITK-SYK-expressing T cells FACS sorted for eGFP^+^ from the spleens of acutely induced *ITK-SYK*^*CD4-creERT2*^ mice. As indicated in (**d**), the mice received anti-PD-L1 or isotype control antibodies on days one, three and five via intraperitoneal injection (n = 3 biological replicates per group). Data was normalized using total cellular protein. ECAR, extracellular acidification rate. P=two-sided Student’s t-test. The data are shown as box plots, in which the middle line denotes the median, the top and bottom box edges denote the 0.25 and 0.75 quantiles, respectively, and the whiskers denote the minimum and maximum values. **f**, OCR metabolic flux analysis from the same experiment as in (**e**) OCR, oxygen consumption rate. P=two-sided Student’s t-test. **b**, Representative data from a single mouse within the experiment shown in Fig. 2c, d. **c**, Representative results from 5 independent experiments with three biological replicates per group. **e,f**, Representative data from two independent experiments with three biological replicates per group. Source data

Based on these findings, we next performed direct ex vivo functional analyses of glucose metabolism of acute ITK–SYK-expressing T cells with and without PD-1 function. Upon oncogenic T cell signaling, we detected enhanced glucose uptake in PD-1-deficient T cells compared to that of wild-type cells (Extended Data Fig. 1b; *P* = 0.0017). Moreover, extracellular flux analysis revealed a greater extracellular acidification rate (ECAR) in transformed ITK–SYK^+^PD-1^−^ T cells than in their premalignant ITK–SYK^+^PD-1^+^ T cell counterparts, whereas the oxygen-consumption rate (OCR) remained unchanged (Fig. 1g,h; *P* < 0.0001 and *P*, not significant (NS)). Because the increase in ECAR implies aerobic glycolytic conversion of pyruvate to lactate, we next measured the production of this metabolite. Lactate generation was enhanced in the transformed ITK–SYK^+^PD-1^−^ T cells compared to in their premalignant counterparts (Fig. 1i; *P* = 0.034). To study glucose metabolism in vivo, we used the glucose analog tracer [^18^F]fluorodeoxyglucose ([^18^F]FDG) and positron emission tomography (PET). The maximum [^18^F]FDG uptake in individual mice was measured in the spleens of both *ITK-SYK*^CD4-CreERT2^ and *ITK-SYK*^CD4-CreERT2^;*Pdcd1*^−/−^ mice (Fig. 2a and Extended Data Fig. 2a). The overall [^18^F]FDG uptake and maximum uptake in single voxels was significantly higher in *ITK-SYK*^CD4-CreERT2^;*Pdcd1*^−/−^ animals than in PD-1-proficient *ITK-SYK*^CD4-CreERT2^ mice, demonstrating augmented glucose uptake in vivo (Fig. 2b; *P* = 0.005). To measure glycolytic conversion, we infused hyperpolarized [1-^13^C]pyruvate intravenously into tamoxifen-injected animals and compared [1-^13^C]lactate/[1-^13^C]pyruvate signal ratios in the spleens using hyperpolarized ^13^C magnetic resonance spectroscopic imaging (MRI)^16^. The [1-^13^C]lactate/[1-^13^C]pyruvate signal ratio was substantially higher in oncogene-expressing *ITK-SYK*^CD4-CreERT2^;*Pdcd1*^−/−^ animals than in *ITK-SYK*^CD4-CreERT2^ mice, confirming enhanced lactate production in vivo (Fig. 2c,d and Extended Data Fig. 2a,b; *P* = 0.0284).

Altogether, these sets of genetically controlled in vivo and ex vivo experiments demonstrate that deficiency in PD-1 forces T cells with oncogenic signaling to adapt their metabolism to aerobic glycolysis. This is characteristic of the Warburg effect that promotes the initiation and progression of most cancer types^17–19^.

### PD-1 represses mTOR and HIF1α in premalignant cells

To determine how PD-1 inactivation promotes glycolysis, we acutely blocked PD-1 function in ITK–SYK-expressing T cells with anti-programmed cell death ligand 1 (PD-L1) checkpoint inhibitors as experimental tools in vivo or ex vivo. This strategy allowed us to control the inactivation of PD-1 signaling in oncogene-expressing T cells in a time-dependent manner. Similar to genetic deletion of *Pdcd1*, injection of anti-PD-L1 antibody into tamoxifen-treated *ITK-SYK*^CD4-CreERT2^ mice induced unrestricted expansion of oncogene-expressing T cells, which rapidly killed the host (Extended Data Fig. 2c)^12^. These acutely PD-1-inhibited cells also switched to glycolysis with an increase in ECAR and no change in their basal OCR (Extended Data Fig. 2d–f; *P* < 0.0001 and *P*, NS). Intracellular flow cytometric analysis directly after acute PD-1 blockade demonstrated that inactivation of the PD-1 signal resulted in prompt activation of the AKT and mTOR pathways within oncogene-expressing T cells (Fig. 2e) and in direct upregulation of HIF1α expression^20^. Furthermore, we observed an induction of HIF1α transcriptional targets, including the glucose transporter GLUT1 and hexokinase 2, which are rate-limiting factors for glucose uptake and glucose utilization, respectively, in normal and malignant lymphocytes (Fig. 2e)^21^. Thus, these key metabolic switches in premalignant cells are under the direct negative control of PD-1 tumor-suppressor signaling.

**Extended Data Fig. 3:**
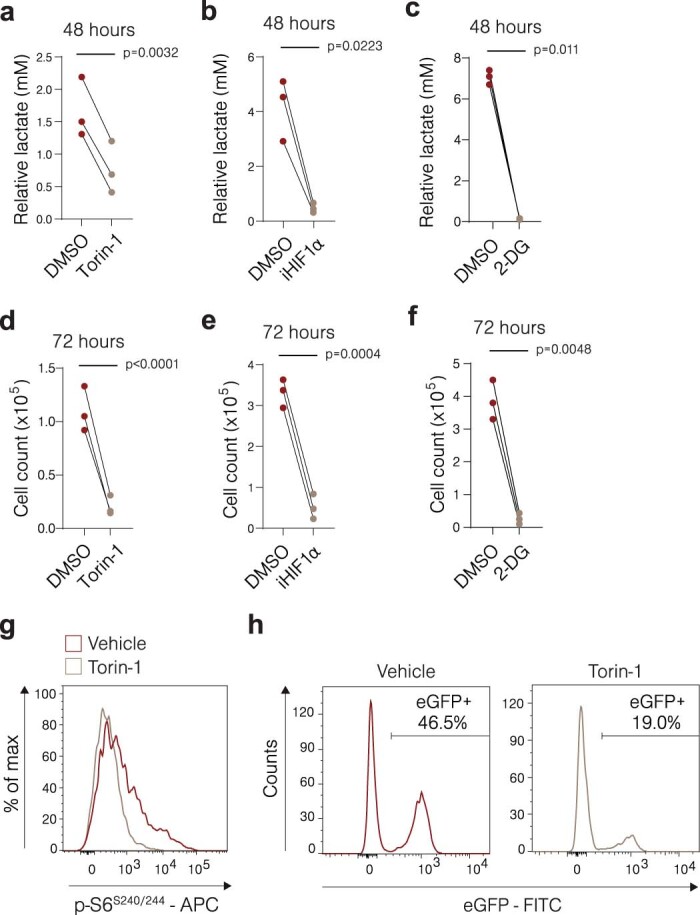
Transformed T cells with *Pdcd1* deletion depend on the mTOR-HIF1α-glycolysis cascade. **a,b,c**, Lactate concentrations in the cell culture supernatants of eGFP^+^ FACS sorted cells derived from the spleens of acutely induced *ITK-SYK*^*CD4-creERT2*^*;Pdcd1*^−/−^ mice and incubated *in vitro* for 48 h in the presence of the mTOR inhibitor Torin-1 (**a**; 0.1 μM), HIF1α inhibitor PX-478 (**b**; iHIF1α, 5 μM) or glycolysis inhibitor 2-DG (**c**; 1 mM) or DMSO as a control (n = 3 biological replicates). Lactate concentrations were normalized to the viable cell numbers. P=paired two-sided Student’s t-test. **d,e,f**, Number of viable ITK-SYK^+^PD-1^-^ cells cultured *in vitro* for 72 h with the mTOR inhibitor Torin-1 (**d**; 0.1 μM), HIF1α inhibitor PX-478 (**e**; iHIF1α, 5 μM) or glycolysis inhibitor 2-DG (**f**; 1 mM) or DMSO as a control (n = 3 biological replicates). eGFP^+^ T cells were sorted by FACS from acutely induced *ITK-SYK*^*CD4-creERT2*^*;Pdcd1*^−/−^ animals (n = 3 biological replicates). P=paired two-sided Student’s t-test. **g**, Phosflow analysis of serine 240/244 of S6 ribosomal protein in ITK-SYK^+^PD-1^-^ cells isolated from NSG mice treated with Torin-1 or vehicle. Cells were fixed and permeabilized immediately after isolation. **h**, eGFP percentage of viable lymphocytes in the peripheral blood of Torin-1 or vehicle-treated NSG mice, which were transplanted with 1,000 eGFP^+^ FACS-sorted ITK-SYK^+^PD-1^-^ cells isolated from acutely induced *ITK-SYK*^*CD4-creERT2*^*;Pdcd1*^−/−^ mice. Blood was taken seven days after initiation of treatment. **a,b,c,d,e,f**, Representative results from 2 independent experiments with three biological replicates per group. **g**, Representative results from two out of the six animals analyzed. **h**, Representative results from two out of the thirteen animals analyzed. Source data

To test the dependency of fully transformed lymphomas with *Pdcd1* deletion on the released mTOR–HIF1α–glycolysis cascade, we incubated ITK–SYK^+^PD-1^−^ cells with small-molecule inhibitors of mTOR, HIF1α or glycolysis^22,23^. Direct inhibition of glycolysis with 2-deoxy-d-glucose (2-DG) reduced the production of lactate, similar results were observed with the inhibition of mTOR or HIF1α (Extended Data Fig. 3a–c), and all three treatments were toxic to PD-1-deficient lymphoma cells (Extended Data Fig. 3d–f). Moreover, treatment of ITK–SYK^+^PD-1^−^ lymphoma-bearing mice with the mTOR inhibitor torin-1, which blocks mTOR activity within tumor cells in vivo (Extended Data Fig. 3g), significantly prolonged survival (Fig. 2f and Extended Data Fig. 3h; *P* = 0.0057). Together, these pharmacological studies demonstrate that glycolytic reprogramming is key for the transformation and survival of oncogene-expressing PD-1-deficient T cells.

### PD-1 limits oncogene-triggered energy metabolism

**Fig. 3:**
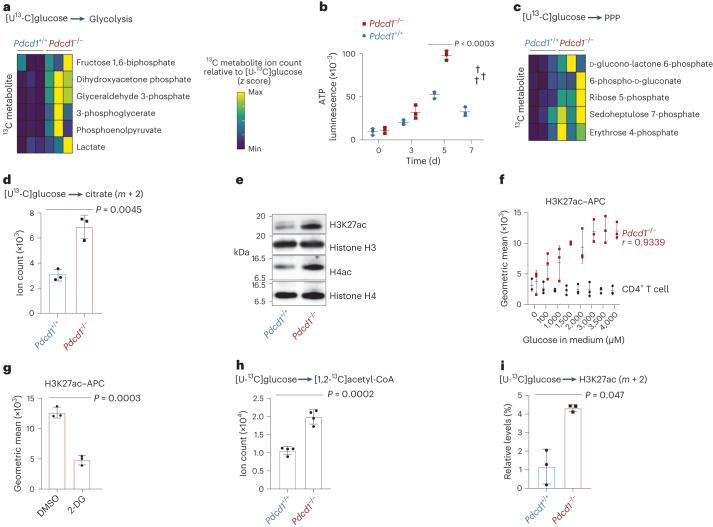
PD-1 facilitates glycolysis-dependent histone acetylation. **a**, Intracellular ^13^C abundance within the glycolysis pathways detected by targeted LC–MS/MS relative to intracellular [U-^13^C]glucose in ITK–SYK^+^ T cells with or without PD-1, cultured with uniformly labeled [U-^13^C]glucose for 3 h in vitro. Data are normalized to viable cell number and *z* scores per metabolite. Min, minimum. **b**, Time-dependent ATP measurement in ITK–SYK^+^ T cells with or without PD-1. *P*, two-sided Student’s *t*-test. Data are presented as the mean ± s.e.m. and individual data points for each animal. The cross indicates death of the animal. **c**, Ion count for intracellular ^13^C-labeled metabolites in the pentose phosphate pathway (PPP). **d**, Ion count for intracellular ^13^C-citrate (*m* + 2) isotopomers. *P*, two-sided Student’s *t*-test. Shown are the mean ± s.d. and individual data points. **e**, Western blot analysis of acetylated histones in ITK–SYK^+^ T cells with or without PD-1. H4ac, H4 acetylation. **f**, Flow cytometry for H3K27ac in wild-type CD4^+^ or ITK–SYK^+^ T cells after a 4-h in vitro incubation with different glucose concentrations. *r*, Pearson’s correlation coefficient. Data are presented as mean ± s.d. and individual data points. **g**, Flow cytometry for H3K27ac in ITK–SYK^+^PD-1^−^ cells after a 4-h in vitro culture with 2-DG (1 mM) or DMSO. *P*, paired two-sided Student’s *t*-test. Data are presented as mean ± s.d. **h**, Levels of [U-^13^C]glucose-derived intracellular [1,2-^13^C]acetyl-CoA determined by targeted LC–MS/MS in ITK–SYK^+^ T cells with or without PD-1 cultured with uniformly labeled [U-^13^C]glucose (11 mM) for 4 h in vitro (*n* = 4 mice per genotype). Data were normalized as in **a**. *P*, two-sided Student’s *t*-test. Data are presented as mean ± s.e.m. **i**, Levels of [U-^13^C]glucose-derived ^13^C-H3K27ac (*m* + 2) determined by orbitrap LC–MS/MS. Experiment as in **h** (with *n* = 3 mice per genotype). Data were normalized as in **a**. *P*, two-sided Student’s *t*-test. Data are presented as mean ± s.d. **a**–**d**, Representative data from two independent experiments with *n* = 3 biological replicates per group. **e**, Representative data from two independent experiments with two biological replicates per group. **f**, Representative data from two independent experiments. **g**, Representative data from two independent experiments with three biological replicates per group. **h**, Representative data from one experiment with four biological replicates per group. **i**, Representative data from one experiment with three biological replicates per group. Source data

In general, cancer cells use the metabolism of glucose to generate ATP for energy supply, rapidly assimilate biomass and generate signaling molecules that can regulate gene expression^18^. To identify glycolysis-dependent lymphoma-enabling mechanisms in an unbiased manner, we incubated acute oncogene-expressing ITK–SYK^+^PD-1^−^ T cells and their premalignant ITK–SYK^+^PD-1^+^ counterparts with uniformly labeled [U-^13^C]glucose ex vivo and performed LC–MS/MS analysis to trace [U-^13^C]glucose-derived metabolites^24^. The amount of ^13^C-labeled glycolysis intermediates, fructose 1,6-bisphosphate, dihydroxyacetone phosphate, glyceraldehyde 3-phosphate, 3-phosphoglycerate, phosphoenolpyruvate and lactate, was elevated in PD-1-deficient lymphoma cells (Fig. 3a), indicating enhanced glucose usage within the canonical upper glycolytic pathway. This pathway is particularly important for energy production^25^. Indeed, inactivation of PD-1 signaling boosts ATP levels (Fig. 3b; *P* < 0.0003), indicating that loss of PD-1 function can overcome the energy deficit in premalignant T cells that is required to fuel overt malignancy.

In addition, we detected increased glucose utilization in the pentose phosphate pathway in ITK–SYK^+^PD-1^−^ lymphoma cells, with augmented generation of [U-^13^C]glucose-derived glucono-lactone 6-phosphate, 6-phospho-d-gluconate, ribose 5-phosphate, sedoheptulose 7-phosphate and erythrose 4-phosphate (Fig. 3c). The overall abundance of [U-^13^C]glucose-derived tricarboxylic acid cycle (TCA) metabolites only slightly differed between transformed ITK–SYK^+^PD-1^−^ T cells and their premalignant ITK–SYK^+^PD-1^+^ counterparts, although the specific levels of citrate (*m* + 2) showed a more than twofold increase in the absence of PD-1 (Fig. 3d; *P* = 0.0045).

### PD-1 facilitates glycolysis-dependent histone acetylation

**Extended Data Fig. 4:**
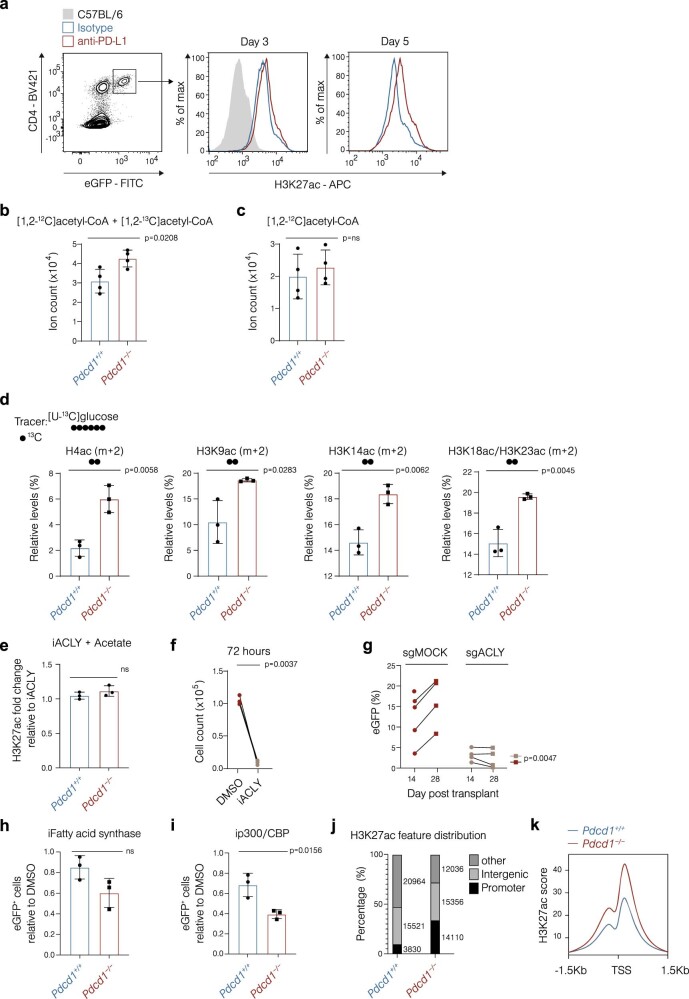
PD-1 controls ACLY-dependent epigenetic reprogramming. **a**, H3K27ac in CD4 + GFP + T cells of *ITK-SYK*^*CD4-creERT2*^ or *C57BL/6* mice on d5 after TAM +/− anti-PD-L1 antibody treatment. **b, c**, Non-labeled (**b**) and labeled (**c**) acetyl-CoA determined by targeted LC-MS/MS in ITK-SYK^+^ T cells +/− PD-1 cultured with uniformly labeled [U-^13^C]glucose (11 mM) for 4 h *in vitro* (n = 4 mice genotype). Normalization to viable cell numbers. P=two-sided Student’s t-test; mean ± SEM. **d**, [U-^13^C]glucose-derived ^13^C-acetyl groups on histones determined by orbitrap LC-MS/MS in ITK-SYK^+^ T cells +/− PD-1 cultured with uniformly labeled [U-^13^C]glucose (11 mM) for 4 h *in vitro* (n = 3 mice genotype). Normalization as in (**b**, **c**). P=two-sided Student’s t-test; mean ± SD. **e**, H3K27ac in ITK-SYK^+^ T cells +/− PD-1 after 3 h *in vitro* incubation with BMS-303141 (‘iACLY’, 15 μM) +/− acetate (100 μM; n = 3 biological replicates). P=paired two-sided Student’s t-test; mean ± SD. **f**, Viable ITK-SYK^+^PD-1^-^ cells after 72 h *in vitro* culture with BMS-303141 (‘iACLY,’ 5 μM) or DMSO (n = 3 biological replicates). P=paired two-sided Student’s t-test. **g**, Viable ITK-SYK^+^ cells in the blood of sgACLY or sgMOCK-injected mice (n = 4 per group). P=two-sided Student’s t-test. **h**, Viable ITK-SYK^+^ cells +/− PD-1 after 3-day *in vitro* incubation with FT113 (1 μM) or DMSO (n = 3 biological replicates). P=two-sided Student’s t-test; mean ± SD. **i**, Viable ITK-SYK^+^ cells in presence of NEO2734 (50 nM). Experiment as in (**e**). P=two-sided Student’s t-test; mean ± SD. **j**, Percentage of H3K27ac ChIP-seq peak distribution over different genomic features as determined from the experiment in (**d**). **k**, Genome-wide average H3K27ac scores in a 3 Kb window around the TSS. ChIP-seq was performed as indicated in Fig. 4**h**. ChIP-seq, chromatin immunoprecipitation sequencing; TSS, transcription start site. **a**, Representative data from two independent experiments with n = 3 biological replicates per genotype. **b**, **c**, one experiment, n = 4 biological replicates. **d**, Data from one experiment. **e**, Representative data from one experiment with n = 3 biological replicates per genotype. **f**, Representative data from two independent experiments. **g**, Data from a single experiment. **h,i**, Representative data from three independent experiments with n = 3 biological replicates per genotype. **j,k**, Data from one experiment. Source data

There is increasing evidence that metabolite abundance is also highly relevant for regulation of the tumor epigenome^26,27^. In particular, histone acetylation is extremely sensitive to the availability of extramitochondrial acetyl-CoA^28^. In this context, glucose-derived citrate, after export from the mitochondrion, is converted by ACLY to oxalacetate and cytosolic acetyl-CoA, which is subsequently used by acetyltransferases to mediate the acetylation of target proteins. After entering the nucleus, this pool of acetyl-CoA is critical for histone acetyltransferases to mediate histone acetylation for chromatin opening^29,30^. Because these pathways can link glucose metabolism to epigenetic regulation, which is frequently altered in T cell malignancy^1^, we next studied the effects of PD-1 inactivation on histone acetylation in ITK–SYK oncogene-expressing primary CD4^+^ T cells. Intriguingly, upon oncogenic T cell signaling, both the genetic deletion of *Pdcd1* and acute pharmacological blockade of PD-1 triggered a significant increase in histone H4 and H3 lysine 27 (H3K27) acetylation (H3K27ac; Fig. 3e and Extended Data Fig. 4a). Ex vivo incubation of ITK–SYK^+^PD-1^−^ lymphoma cells with glucose at increasing concentrations demonstrated that the level of H3K27ac depends directly on glucose availability (Fig. 3f; *r* = 0.9339)^31^. Conversely, inhibition of glycolysis with 2-DG resulted in a reduction in H3K27ac in PD-1-deficient lymphoma cells (Fig. 3g; *P* = 0.0003)^32^, demonstrating a link between PD-1 inactivation and glucose-dependent histone acetylation in oncogene-expressing T cells.

To explore whether this link involves glycolysis-dependent de novo generation of acetyl-CoA, we directly incubated ITK–SYK-expressing T cells from tamoxifen-injected *ITK-SYK*^CD4-CreERT2^ and *ITK-SYK*^CD4-CreERT2^;*Pdcd1*^−/−^ mice ex vivo with [U-^13^C]glucose and studied acetyl-CoA production by targeted LC–MS/MS analysis. Indeed, in the transformed ITK–SYK^+^PD-1^−^ T cells, there was a significant increase in [1,2-^13^C]acetyl-CoA and total acetyl-CoA ([1,2-^12^C]acetyl-CoA + [1,2-^13^C]acetyl-CoA) generation (Fig. 3h and Extended Data Fig. 4c; *P* = 0.0002 and *P* = 0.0206), whereas the levels of [1,2-^12^C]acetyl-CoA did not differ (Extended Data Fig. 4b; *P*, NS). Next, we isolated histones from these oncogene-expressing primary T cells incubated with [U-^13^C]glucose for high-resolution LC–MS/MS analysis. We observed higher de novo acetylation of histones H3 and H4 with [U-^13^C]glucose-derived [^13^C]acetyl marks at H3K27, H3 lysine 9 (H3K9), H3 lysine 14 (H3K14), H3 lysine 18 (H3K18) and H3 lysine 23 (H3K23) in transformed ITK–SYK^+^PD-1^−^ T cells than in PD-1-proficient ITK–SYK^+^PD-1^+^ cells (Fig. 3i and Extended Data Fig. 4d; *P* = 0.047)^33^. These data indicate that histone acetylation requires glycolysis-dependent acetyl-CoA synthesis after PD-1 inactivation in T cells with oncogenic T cell signaling.

### ACLY is a critical effector molecule downstream of PD-1

**Fig. 4:**
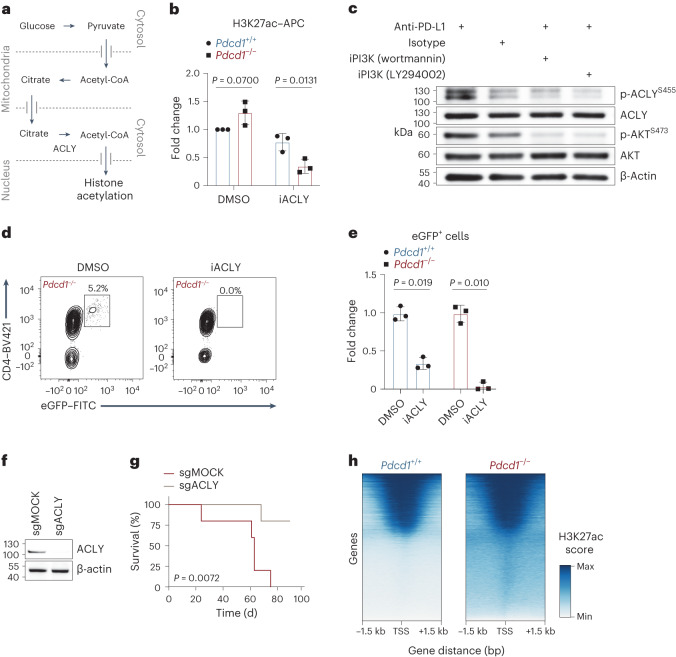
ACLY is a critical effector molecule downstream of PD-1. **a**, Schematic of glucose flux to histone acetyl groups. **b**, Flow cytometry for H3K27ac in ITK–SYK^+^ cells after 4 h of in vitro culture with the ACLY inhibitor BMS-303141 (‘iACLY’, 15 μM) or DMSO. ITK-SYK^+^ cells were sorted by flow cytometry from *ITK-SYK*^CD4-CreERT2^ mice on day 5 after tamoxifen injection and 12 h of anti-PD-L1 or isotype control antibody treatment (intraperitoneal, 200 μg). *P*, paired two-sided Student’s *t*-test. Data are presented as the mean ± s.d. **c**, Western blot analysis in lysates from ITK–SYK^+^PD-1^+^ cells. Spleen-derived single-cell suspensions were sorted by flow cytometry from *ITK-SYK*^CD4-CreERT2^ mice on day 5 after tamoxifen injection and cultured in vitro overnight with anti-PD-L1 or isotype control antibodies and the PI3K inhibitors (‘iPI3K’) wortmannin (20 nM), LY294002 (20 μM) or DMSO. **d**, Flow cytometry for CD4 and eGFP in viable lymphocytes derived from spleens of acutely induced *ITK-SYK*^CD4-CreERT2^;*Pdcd1*^−/−^ mice after 3 d of in vitro culture with BMS-303141 (5 μM) or DMSO. **e**, Fold change in viable ITK–SYK-expressing eGFP^+^ cells in medium supplemented with BMS-303141 (5 μM) or DMSO. Spleen-derived lymphocytes, sorted by flow cytometry from *ITK-SYK*^CD4-CreERT2^ and *ITK-SYK*^CD4-CreERT2^;*Pdcd1*^−/−^ mice on day 5 after tamoxifen injection, were incubated for 3 d in vitro. *P*, two-sided Student’s *t*-test. Data are presented as mean ± s.d. **f**, Western blot for ACLY in ITK–SYK^+^PD-1^−^ cells electroporated with *Acly-*targeting (sgACLY) or non-targeting control (sgMOCK)RNP complexes. Protein lysates were generated after 48 h of in vitro culture. **g**, Survival of recipient mice transplanted with *Acly* (‘sgACLY’) or mock-edited (‘sgMOCK’) ITK–SYK^+^PD-1^−^ cells. *P*, two-sided log-rank test. **h**, Gene-wise global H3K27ac scores from anti-H3K27ac ChIP–seq in a 1.5-kb window around the TSS. ITK–SYK^+^ cells were sorted by flow cytometry from *ITK-SYK*^CD4-CreERT2^ and *ITK-SYK*^CD4-CreERT2^;*Pdcd1*^−/−^ mice on day 5 after tamoxifen injection, fixed and processed for anti-H3K27ac ChIP–seq analysis. **b**, Representative data from three independent experiments with three biological replicates per group. **c**, Representative data from two independent experiments with two biological replicates. **d**,**e**, Representative data from three independent experiments. **f**,**g**, Data from one experiment with *n* = 5 mice per group. **h**, Data from a single experiment with two biological replicates per group. Source data

As indicated above, ACLY is the key enzyme that mediates the generation of glucose-derived extramitochondrial acetyl-CoA (Fig. 4a)^34^. To explore the function of ACLY in PD-1-regulated histone acetylation, we treated tamoxifen-injected *ITK-SYK*^CD4-CreERT2^ mice with anti-PD-L1 or isotype control antibodies and isolated oncogene-expressing CD4^+^ T cells. Next, we incubated these cells in vitro with the small-molecule ACLY inhibitor BMS-303141 for 3 h^32^. This treatment reduced histone acetylation in ITK–SYK^+^ cells in comparison to the dimethyl sulfoxide (DMSO) vehicle control (Fig. 4b). Notably, the dependence of H3K27ac on ACLY function was significantly higher in ITK–SYK-expressing cells with pharmacologically inactivated PD-1 pathway (Fig. 4b; *P* = 0.0131). Consistent with published results, exogenous supplementation of acetate at physiological levels (100 µM) could not restore decreased H3K27ac signals in ACLY inhibitor-treated lymphoma cells (Extended Data Fig. 4e).

The activity of ACLY itself can in principle be regulated by AKT, which can phosphorylate ACLY at serine 455, a key point of control for this enzyme^35–39^. As PD-1 inactivation results in (PI3K)–AKT signaling in oncogene-expressing T cells (Fig. 2e), we hypothesized that these events could additionally trigger ACLY activation^39^. To test this hypothesis, we induced ITK–SYK expression in CD4^+^ cells by injecting tamoxifen into *ITK-SYK*^CD4-CreERT2^ mice and acutely blocked PD-1 function with anti-PD-L1 treatment. Inactivation of PD-1 signaling resulted in direct activation of ACLY, as measured by phosphospecific phosphorylated (p)-ACLY^S455^ antibodies (Fig. 4c), and this phosphorylation could be reversed by PI3K inhibition with wortmannin or LY294002 (Fig. 4c)^40^.

To determine whether ACLY activity is required for PD-1-deficient lymphoma cell survival, we next incubated transformed ITK–SYK^+^PD-1^−^ T cells from tamoxifen-injected *ITK-SYK*^CD4-CreERT2^;*Pdcd1*^−/−^ mice with an ACLY inhibitor. This treatment killed ITK–SYK^+^PD-1^−^ lymphoma cells (Extended Data Fig. 4f; *P* = 0.0037). Importantly, co-cultures with splenocytes demonstrated that ITK–SYK^+^PD-1^−^ T cells were highly sensitive to ACLY inhibition, whereas ITK–SYK^+^PD-1^+^ T cells and normal T cells were less affected (Fig. 4e,f; *P* = 0.019 and *P* = 0.010).

To genetically define whether ACLY is necessary for PD-1-deficient lymphoma growth in vivo, we disrupted *Acly* in transformed ITK–SYK^+^PD-1^−^ T cells using CRISPR–Cas9-mediated gene editing and transplanted these cells into syngeneic wild-type hosts (Fig. 4f). While the control-edited ITK–SYK^+^PD-1^−^ cells proliferated rapidly in vivo, as measured by eGFP monitoring, and killed all recipient animals as expected in less than 80 d, ACLY-deficient ITK–SYK^+^PD-1^−^ T cells were unable to expand (Extended Data Fig. 4g and Fig. 4i; *P* = 0.0072). The acetyl-CoA pool can be used for both histone acetylation and other acetyl-CoA dependent pathways such as fatty acid synthesis. To distinguish the importance of these pathways, we used pharmacological inhibitors. Our experiments demonstrated that ITK–SYK^+^PD-1^−^ cells were more sensitive to direct inhibition of histone acetylation with inhibitors of histone acetyltransferases p300 and CBP than their PD-1-competent counterparts. By contrast, inhibition of fatty acid synthase did not reveal a significant difference (Extended Data Fig. 4h,i; *P* = 0.0156).

Together, these functional and genetic data demonstrate that loss of PD-1 tumor-suppressor function results in enhanced ACLY activity via enforced (PI3K)–AKT signaling and that this mechanism is critical for both glucose-dependent histone acetylation and the malignant expansion of PD-1-deficient lymphomatous T cells.

### PD-1 controls epigenetic reprogramming and AP-1 activity

**Fig. 5:**
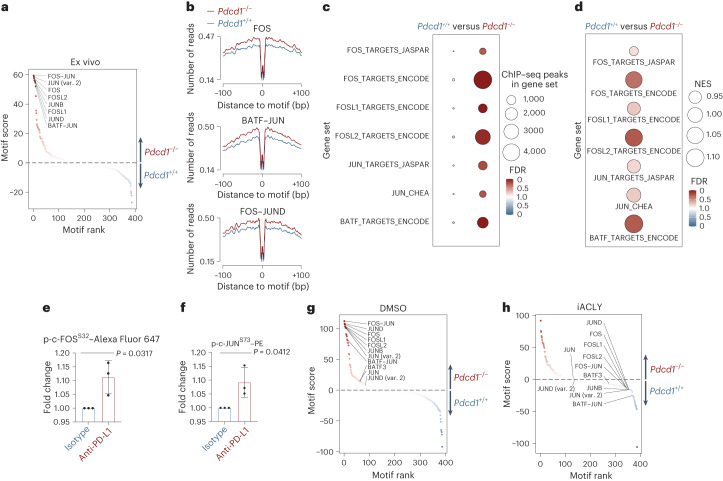
PD-1 controls AP-1 activity in an acetyl-CoA-dependent manner. **a**, Differential transcription factor footprint analysis of ATAC-seq data. ITK–SYK^+^ cells were sorted by flow cytometry from *ITK-SYK*^CD4-CreERT2^ and *ITK-SYK*^CD4-CreERT2^;*Pdcd1*^−/−^ mice on day 5 after tamoxifen injection and immediately processed for ATAC-seq. JASPAR database motif names for the selected AP-1 family transcription factors are indicated. Enrichment of a transcription factor motif is indicated by motif score, motif rank and color. **b**, Binding profiles of selected transcription factors for the indicated genotypes from the experiment shown in **a**. **c**, ChIP-Enrich analysis of ChIP–seq data shown in Fig. 4h for the indicated gene sets. FDR, color intensity of the circles; ChIP–seq peaks in gene set, circle diameter. Blue and red indicate the genotype in which a signature was positively enriched. **d**, GSEA for the indicated gene sets. FDR, color intensity of circles; NES, circle diameter. Blue and red indicate the group in which a signature was positively enriched. **e**, Phosflow analysis of p-c-FOS^S32^ in ITK–SYK^+^ cells. Single-cell suspensions were generated from lymph nodes of *ITK-SYK*^CD4-CreERT2^ mice on day 5 after tamoxifen injection and 12 h of anti-PD-L1 or isotype control antibody treatment (intraperitoneal, 200 μg). *P*, paired two-sided Student’s *t*-test. Data are presented as mean ± s.d. **f**, Phosflow analysis of p-c-JUN^S73^. Same experiment as in **e**. **g**,**h**, Differential transcription factor footprint analysis of ATAC-seq data from ITK–SYK^+^PD-1^+^ and ITK–SYK^+^PD-1^-^ cells after 3 h of in vitro incubation with BMS-303141 (5 μM, **h**) or DMSO (**g**). Spleen-derived eGFP^+^ cells were sorted by flow cytometry from *ITK-SYK*^CD4-CreERT2^ and *ITK-SYK*^CD4-CreERT2^;*Pdcd1*^−/−^ mice on day 5 after tamoxifen injection, incubated in iACLY- or DMSO-containing medium and processed for ATAC-seq. Legend as in **a**. **a**,**b**, Data from a single experiment with three biological replicates per genotype. **c**, Data from one experiment with two biological replicates per group. **d**, Data from a single experiment with four biological replicates per genotype. **e**,**f**, Representative data from two independent experiments with three biological replicates per group. **g**,**h**, Data from one experiment with three biological replicates per genotype. var., variant. Source data

After establishing enhanced histone acetylation as a consequence of *Pdcd1* inactivation in oncogene-expressing T cells, we next tested the epigenetic effects of this mechanism on genome regulation using genome-wide assays. Because H3K27 showed the greatest dependence on glucose-dependent de novo acetylation, we immunoprecipitated H3K27-acetylated histones from ITK–SYK-expressing T cells with or without PD-1 activity and performed high-throughput chromatin immunoprecipitation followed by sequencing (ChIP–seq^41^). Overall, deletion of *Pdcd1* increased H3K27ac in oncogene-expressing T cells, particularly within promoter regions and around gene transcription start sites (TSS), indicating direct effects on transcriptional regulation (Fig. 4h and Extended Data Fig. 4j,k)^42,43^. To assess how these events would affect chromatin accessibility for transcription factor occupancy, we conducted a genome-wide assay for transposase-accessible chromatin using sequencing (ATAC-seq^44^) and performed transcription factor footprint analysis^45–47^. Intriguingly, in PD-1-deficient ITK–SYK-expressing T cells, we observed increased activity scores for the AP-1 family transcription factors c-FOS, FOSL1, FOSL2, c-JUN, JUNB and BATF (Fig. 5a,b). Next, we tested whether the histones at the AP-1 family target gene sequences are hyperacetylated in PD-1-deficient ITK–SYK-expressing T cells by performing GSEA using the H3K27ac ChIP–seq dataset (Fig. 4h; ChIP-Enrich analysis^48^) and the transcription factor target gene sets from the ENCODE, JASPAR and ChEA databases^46,49–51^. We detected a significant enrichment of c-FOS, FOSL1, FOSL2, c-JUN, and BATF target genes in H3K27ac ChIP–seq peaks upregulated in ITK–SYK^+^PD-1^−^ lymphoma cells compared to their premalignant ITK–SYK^+^PD-1^+^ counterparts (Fig. 5c). Furthermore, global expression of AP-1 target mRNA species also increased with positive enrichment of the c-FOS, FOSL1, FOSL2, c-JUN and BATF gene sets in transformed ITK–SYK^+^PD-1^−^ cells (Fig. 5d).

To investigate the underlying mechanism for AP-1 hyperactivation, we focused on the top-ranking motif in PD-1-deficient lymphoma cells, which was the c-FOS–c-JUN heterodimer (Fig. 5a). We performed additional ex vivo experiments to determine the PD-1-dependent phosphorylation status of c-FOS at serine 32 and of c-JUN at serine 73, respectively. These post-translational modifications are known to increase protein stability, nuclear localization and transcriptional activity^52,53^. To capture immediate changes upon PD-1 pathway inactivation, we used pharmacological blockade of PD-1 as introduced above (Fig. 2e). Our Phosflow data revealed a significant increase in p-c-FOS^S32^ and p-c-JUN^S73^ signals in ITK–SYK-expressing cells isolated from animals that had been treated with anti-PD-L1 checkpoint inhibition, suggesting that PD-1 indeed controls the transduction of oncogenic signals to AP-1 family members (Fig. 5e,f; *P* = 0.0317 and *P* = 0.0412).

We asked whether AP-1 hyperactivity in ITK–SYK^+^PD-1^−^ lymphoma cells is induced by ACLY activity and subsequently inflated acetyl-CoA pools. To address this question, we again isolated ITK–SYK^+^PD-1^+^ and ITK–SYK^+^PD-1^−^ cells from tamoxifen-treated *ITK-SYK*^CD4-CreERT2^ and *ITK-SYK*^CD4-CreERT2^;*Pdcd1*^−/−^ animals. This time, we incubated the cells in vitro in the presence of the ACLY inhibitor BMS-303141 or DMSO for 3 h. Next, we sorted eGFP^+^ cells by flow cytometry and performed ATAC-seq and transcription factor footprint analysis. In agreement with the ex vivo results (Fig. 5a), we detected AP-1 family members as top motifs enriched in PD-1-deficient cells (Fig. 5g). Strikingly, upon pharmacological inhibition of ACLY, AP-1 footprints were de-enriched in *Pdcd1*-deleted cells in comparison to their counterparts with intact PD-1 pathway (Fig. 5h).

**Extended Data Fig. 5:**
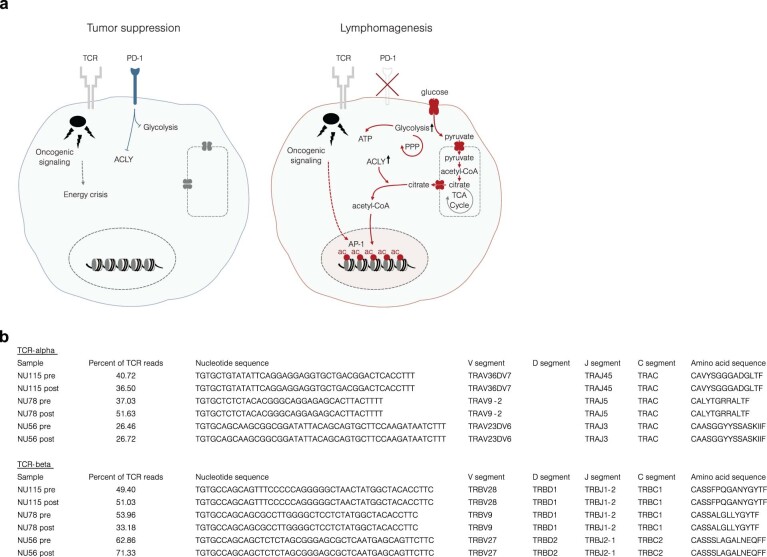
PD-1 tumor suppressor function. **a**, Schematic model**:** Loss of PD-1 tumor suppressor function enables enhanced glucose uptake and glycolytic remodeling upon oncogenic T cell signaling. These events are critical for energy production in pre-malignant cells, for providing building blocks for rapid proliferation and preserving redox balance during T cell transformation and lymphoma progression. In addition, loss of PD-1 signaling unleashes ACLY activity and thereby triggers glucose-dependent production of extramitochondrial acetyl-CoA to mediate the de novo acetylation of histones to open chromatin and enhance the activity of oncogenic AP-1 family transcription factors. **b**, T-cell receptor sequence analysis of three CTCL patients with hyperprogressive disease. The nucleotide sequence, V-D-J-C segments, amino acid sequence, and percentage of T-cell receptor reads are shown for each sample before (‘pre’) and after (‘post’) hyperprogression. **b**, Data from one experiment.

Altogether, these results from our genetically defined mouse models demonstrate that the loss of PD-1 signaling unleashes ACLY activity and thereby triggers glucose-dependent production of extramitochondrial acetyl-CoA to mediate de novo acetylation of histones to open chromatin and enable the enhanced activity of oncogenic AP-1 family transcription factors. Importantly, AP-1 hyperactivation in *Pdcd1*-deleted lymphoma cells was critically dependent on ACLY activity. Thus, our results indicate a link between glucose-derived acetyl-CoA availability and selective opening of compact chromatin at AP-1-binding sites, which is mediated by the PD-1 pathway (for a schematic model, see Extended Data Fig. 5a).

### Glycolysis and AP-1 activation in human *PDCD1*-mutated T-NHL

**Fig. 6:**
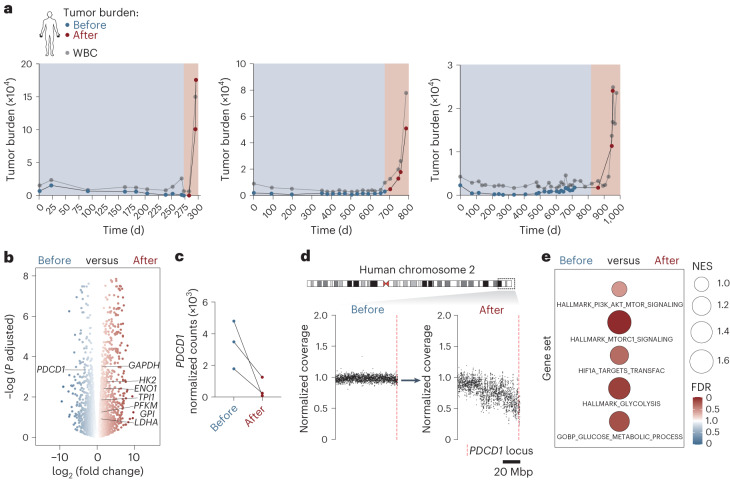
PD-1 inactivation and glycolytic switch in hyperprogressive T-NHLs. **a**, Serial longitudinal analysis of the tumor burden index and white blood cell (WBC) count for three individual patients with CTCL and hyperprogressive disease. Hyperprogression, rapid increase in tumor burden index (‘after’, shaded in red) after an extended period of stable disease course (‘before’, shaded in blue). **b**, Differential gene expression analysis of RNA-seq data derived from the three patients with CTCL in **a** before and after hyperprogression. Data points for selected glycolysis-related genes and *PDCD1* are indicated. *P*, two-sided Wald test. **c**, RNA-seq read count of *PDCD1* transcripts before and after hyperprogression shown for each patient individually. **d**, Whole-genome sequencing-based copy number aberration analysis of *PDCD1-*containing chromosomal region 2q36.3–2q37.3. The time point of genomic DNA isolation relative to hyperprogression is indicated (‘before’ versus ‘after’). The patient shown corresponds to the first patient in **a**. Top: the dashed box indicates the genomic region on human chromosome 2 that is shown in detail at the bottom. Bottom: copy number aberration analysis. The location of the *PDCD1* locus is shown. **e**, GSEA for the indicated gene sets based on RNA-seq data derived from three patients with hyperprogression (**a**). The time point of RNA extraction relative to hyperprogression is indicated (‘before’ versus ‘after’). FDR, color intensity of circles; NES, circle diameter. Blue and red indicate the group in which a signature was positively enriched. **a**, Longitudinally collected data from three individual patients. **b**–**e**, Data from a single experiment with three patients. Source data

To elucidate whether these PD-1-dependent metabolic and epigenetic changes are also present in human T cell lymphoma, we analyzed leukemic stage primary patient samples from cohorts of clinically annotated T-NHLs of cutaneous origin by isolating malignant lymphoma cells by flow cytometry. We first examined a cohort of patients who experienced rapid disease progression, where the blood tumor burden increased by >400% in less than a 1-month period, which we termed ‘hyperprogression’ (Fig. 6a). TCR clonotyping confirmed that post-hyperprogression samples originated from identical T cell clones as the pre-hyperprogression samples (Extended Data Fig. 5b). Intriguingly, in all three instances, hyperprogressive disease was associated with profound downregulation of *PDCD1* transcripts (Fig. 6b,c; *P* = 4.6 × 10^−4^, log_2_ (fold change) = −3.3). Notably, one of these cancers acquired a genetic deletion of *PDCD1* upon disease progression (Fig. 6d), whereas the others lost *PDCD1* expression by still uncharacterized mechanisms. These pre- and post-hyperprogression lymphomas were used for comparative RNA-seq and GSEA analysis. Consistent with our mouse model, we detected a significant enrichment of all signatures that represent enhanced activity of the (PI3K)–AKT–mTOR pathway, enforced HIF1α activity and increased glucose metabolism in post-hyperprogression samples with impaired *PDCD1* expression compared to pre-hyperprogression samples (Fig. 6e).

**Fig. 7:**
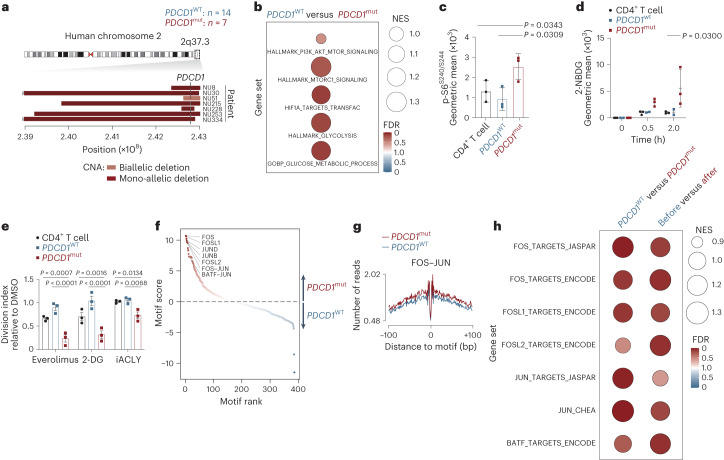
ACLY dependence and AP-1 activation in human PD-1-defective T-NHL. **a**, Genomic region within q37.3 on chromosome 2 in 21 patients with CTCL. CNA, copy number aberrations. *PDCD1*^mut^, *PDCD1* mutant; *PDCD1*^WT^, *PDCD1* wild type. **b**, GSEA for the indicated gene sets based on RNA-seq data from the 21 patients with CTCL shown in **a**. **c**, Phosflow analysis of serine 240 and 244 of S6 ribosomal protein in *PDCD1*-mutant (*n* = 3) and *PDCD1*-wild-type (*n* = 3) primary CTCL cells and in CD4^+^ T cells from healthy donors (*n* = 3). *P*, one-way ANOVA and two-sided Student’s *t*-test. Shown are the mean ± s.d. and individual data points. **d**, Uptake of the glucose analog 2-NBDG in *PDCD1*-mutant (*n* = 3) and *PDCD1*-wild-type (*n* = 3) primary CTCL cells and in CD4^+^ T cells from healthy donors (*n* = 3). Malignant cells sorted by flow cytometry were stimulated with anti-CD3 and anti-CD28 beads, and 2-NBDG uptake was determined at the indicated time points. *P*, two-sided Student’s *t*-test. Shown are the mean ± s.e.m. and individual data points. **e**, Relative division index of malignant T cells from three patients with mutated *PDCD1* or reduced PD-1 expression after hyperprogression compared to three *PDCD1*-wild-type samples and healthy CD4^+^ T cells (*n* = 3) in the presence of 2-DG (1 mM), everolimus (0.1 μM) or BMS-303141 (iACLY, 10 μM). *P*, two-sided Student’s *t*-test. Data are presented as the mean ± s.e.m. and individual data points. **f**, Differential transcription factor footprint analysis of ATAC-seq data from three patients with CTCL with (‘*PDCD1*^mut^’) and three patients without (‘*PDCD1*^WT^’) mutated *PDCD1*. Legend as in Fig. 5a. **g**, Binding profile for the JASPAR FOS–JUN heterodimer motif for the indicated genotypes from the experiment shown in **f**. **h**, GSEA for the indicated gene sets with RNA-seq data from 21 patients with CTCL who were *PDCD1* mutant or *PDCD1* wild type and three patients with hyperprogression. **a**,**b**, Data from a single experiment with 21 patients. **c**,**d**, Data from one experiment with three patients per group and three healthy donors. **e**, Data from one experiment with three patients and three healthy donors. **f**,**g**, Data from one experiment with three patients per group. **h**, Data from a single experiment. Source data

**Extended Data Fig. 6:**
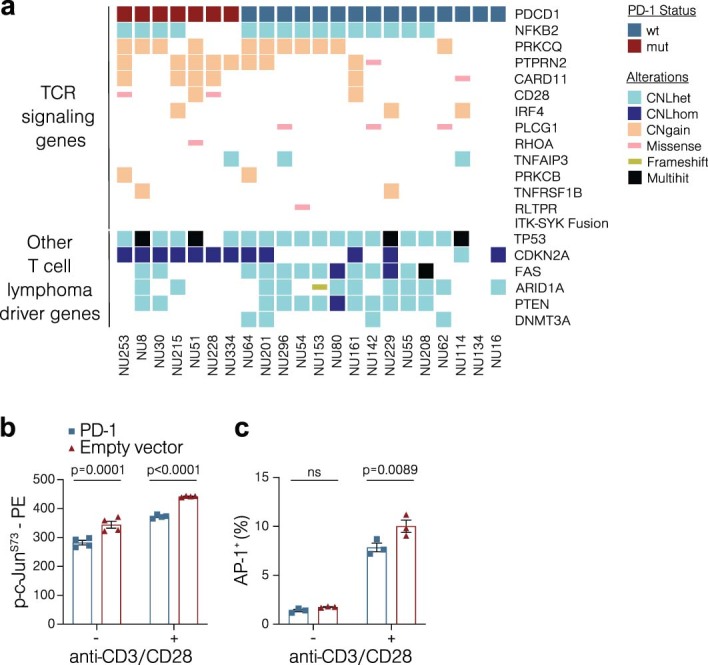
PD-1 controls post-translational AP-1 activation and transcriptional activity. **a**, Oncoplot depicting mutation in T cell receptor (TCR) signaling pathway or other pathways detected in CTCL. Samples are grouped by PD-1 status, either wild-type (‘wt’) or mutant (‘mut’). **b,c**, MFI of p-c-Jun^S73^ (**b**, n = 4) and percentage of cells positive for AP-1 reporter (**c**, n = 3) in Jurkat cells co-cultured with PD-L1 overexpressing Raji cells, with or without anti-CD3/CD28 beads. Jurkat cells were transduced with a lentivirus encoding either PD-1 or an empty vector control. In **b**, cells were co-cultured for 1 hour prior to analysis. In **c**, cells were co-cultured for 24 hours prior to analysis. P = 2-way ANOVA followed by Sidak’s multiple comparison test. MFI, mean fluorescence intensity. The data are presented as the mean ± SEM and individual data points are shown. **a**, Data from one experiment. **b,c**, Representative data from 2 independent experiments. Source data

In addition, we studied a second cohort of patients with cutaneous T cell lymphoma (CTCL) that were either *PDCD1* wild type or *PDCD1* mutant at initial diagnosis. In line with previously reported frequencies^2,12^, we detected *PDCD1* deletions in seven of the 21 specimens (Fig. 7a). RNA-seq and GSEA revealed a significant enrichment of the key gene sets for (PI3K)–AKT–mTOR signaling and HIF1α activation (Fig. 7b) in *PDCD1*-mutant patients. Again, we also observed enhanced activity of glycolysis gene sets in *PDCD1-*mutant lymphomas compared to *PDCD1*-wild-type lymphomas (Fig. 7b). None of the analyzed primary patient samples harbored the rare ITK–SYK fusion oncogene that we used in the mouse to model oncogenic TCR signaling. Instead, *PDCD1*-mutant human tumors harbored diverse and numerous oncogenic TCR signaling mutations including in *CD28*, *PLCG1* and *RHOA*, suggesting that transcriptional effects are not tied to the identity of the oncogene (Extended Data Fig. 6a). Interestingly, multiple *PDCD1*-mutant lymphomas carried mutations in another tumor suppressor encoded by *CDKN2A* (Extended Data Fig. 6a).

Next, we used viable primary patient samples from these two cohorts to functionally assess our findings on the (PI3K)–AKT–mTOR axis, which is under PD-1 control in normal and malignant T cells, and our discovery of ACLY as a PD-1 target molecule in T-NHL. First, we probed the impact of *PDCD1* deletions on (PI3K)–AKT–mTOR signaling and glucose uptake based on six individual patients, three with mutant *PDCD1* and three with wild-type *PDCD1*. In line with our findings in the murine model, *PDCD1*-mutant lymphoma cells exhibited a significant increase in S6 ribosomal protein phosphorylation, demonstrating enhanced mTOR activity compared to *PDCD1*-wild-type cells (Fig. 7c; *P* = 0.0309)^54^. Moreover, uptake of the fluorescent glucose analog 2-NBDG was also significantly increased in *PDCD1*-mutant lymphoma cells (Fig. 7d; *P* = 0.03)^55^. To perturb (PI3K)–AKT–mTOR signaling, glycolysis or ACLY activity in viable *PDCD1*-wild-type and *PDCD1*-mutant lymphoma cells, we incubated the six primary patient samples with respective small-molecule inhibitors of these pathways. Treatment with the mTORC1 inhibitor everolimus, which is currently in clinical trials for T-NHL therapy (NCT00918333 and NCT01075321), reduced viability and division of *PDCD1*-mutant cancer cells (Fig. 7e). Similarly, inhibition of glycolysis with 2-DG or ACLY blockage with BMS-303141 also significantly impaired cell proliferation in PD-1-deficient lymphomas (Fig. 7e). Importantly, stage-matched PD-1-wild-type tumors were resistant to everolimus, 2-DG and inhibition of ACLY. These data suggest that PD-1 loss confers therapeutic vulnerability to these inhibitors (Fig. 7e).

Finally, we assessed chromatin accessibility in primary cells from three *PDCD1*-wild-type and three *PDCD1*-mutant CTCLs by ATAC-seq and performed transcription factor footprint analysis (Fig. 7f,g). Similar to the murine model, the top transcription factor motifs most enriched in the *PDCD1* mutant compared to the wild type were AP-1 family members, including c-FOS, FOSL1, FOSL2, c-JUN, JUNB and BATF, which we corroborated with functional in vitro assays (Fig. 7f,g and Extended Data Fig. 6b,c). Furthermore, at the global transcript level, both *PDCD1*-mutant and post-hyperprogression T-NHL samples showed a significant enrichment in c-FOS, FOSL1, FOSL2, c-JUN and BATF target genes (Fig. 7h).

## Discussion

Here, we identified the key components of the PD-1 tumor-suppressor program in T cell lymphoma. Using a combination of genetic mouse models and primary human T-NHL samples, our results highlight the role of PD-1 as a critical gatekeeper molecule curtailing the glycolytic switch during neoplastic transformation of a T cell. We provide evidence that metabolic reprogramming via the (PI3K)–AKT–mTOR–HIF1α axis leads to induction of rate-limiting factors for glucose uptake and metabolization to enforce tumor cell energy production. This tumor-suppressive activity of PD-1 specifically suppresses glycolytic reprogramming but not oxidative phosphorylation.

Interestingly, our data presented here and previous work^2^ indicate that PD-1-mutation status could serve as a potential biomarker for genotype-specific vulnerabilities in T cell lymphomas. For example, mTOR is currently being evaluated as a target in T-NHL with heterogeneous clinical results^56^. We provide evidence that molecular stratification based on genetic *PDCD1* status could help to identify patients who will likely respond to mTOR pathway inhibition. Moreover, our data suggest enhanced ACLY activity as a selective vulnerability in *PDCD1-*mutant T-NHLs. Furthermore, we describe a previously unknown link between PD-1 signaling and metabolite-controlled epigenetic reprogramming. Upon PD-1 loss, increased pools of glycolysis-derived citrate permit enhanced acetyl-CoA production. This in turn enforces opening of compact chromatin at AP-1-binding sites and enhanced transcription of AP-1 target genes. Conversely, pharmacological inhibition of ACLY curtails AP-1 hyperactivation and is toxic to *PDCD1*-mutant lymphomas. Notably, antigenic stimulation of non-transformed T cells also induces AP-1 activity and enforces AP-1-directed chromatin remodeling^57^. However, in contrast to oncogenic AP-1 activation in T-NHL, this process strictly depends on external induction of co-stimulatory pathways such as CD28 (ref. ^57^) through still ill-defined mechanisms.

While our results identify PD-1 as a metabolic gatekeeper for T-NHL energy metabolism and induction of the epigenetic ACLY–AP-1 axis, several questions remain open. On the one hand, it is unclear whether unleashed ACLY activity in *PDCD1*-mutant lymphomas regulates acetyl-CoA-dependent metabolic pathways apart from histone acetylation, similar to de novo generation of lipids. On the other hand, it is undefined how PD-1 signaling regulates individual AP-1 family members. While our data show enhanced phosphorylation of key serine residues within the transactivation domains of c-JUN and c-FOS in the absence of PD-1 signaling, the precise mechanisms that underlie these effects remain to be uncovered. In this context, it will also be critical to elucidate how acetyl-CoA abundance can selectively enhance recruitment of AP-1 factors to AP-1–DNA binding sites and whether related pathways are also active in non-transformed T cells and during T cell exhaustion, which is frequently associated with chronic PD-1 signaling and dysregulated AP-1 activity^58^.

In sum, our findings establish PD-1 as a central regulator for tumor cell energy metabolism and AP-1 activity in T-NHL. We demonstrate that PD-1 restricts glycolysis, glucose-derived acetyl-CoA production and chromatin remodeling. PD-1-deficient T-NHLs acquire epigenetic changes that increase access for AP-1 factors in otherwise compact DNA. This mechanism is highly conserved between human and murine lymphomas. Our results link PD-1 mutations with AP-1 hyperactivation, which has been demonstrated to be a hallmark of aggressive, drug-resistant T-NHLs^59^. Moreover, our data highlight the (PI3K)–AKT–mTOR–HIF1α–ACLY-axis as a genotype-specific therapeutic vulnerability in aggressive T cell lymphomas with defective PD-1 function, which should be tested in future clinical interventions.

## Methods

We confirm that all experiments with mice and human patient-derived material were performed in accordance with local animal-protection guidelines (Regierung von Oberbayern, Munich, Germany) or after approval by the Northwestern University Institutional Review Board, respectively.

### Mice

Mice of both sexes aged 6–12 weeks were used for all experiments. Littermate controls were used whenever possible. Randomization and blinding were not performed. *ITK-SYK*^CD4-CreERT2^ and *ITK-SYK*^CD4-CreERT2^;*Pdcd1*^−/−^ animals were described earlier and maintained on a C57BL/6 genetic background^12^. *Pdcd1*^−/−^ (028276), B6J.129(Cg)-*Gt(ROSA)26Sor*^tm1.1(CAG-cas9*,-EGFP)Fezh^/J (Cas9, 026179) and NOD.CG-*Prkdc*^*scid*^ *IL2rg*^*tm1Wjl*^/SzJ (NOD–SCID gamma (NSG), 005557) mice were purchased from Jackson Laboratory. All animal experiments were performed in accordance with local guidelines (Regierung von Oberbayern, Munich, Germany). Mice were euthanized if they exhibited signs of lymphoma (lymph node enlargement, palpable tumor, labored breathing, ascites) or if they lost 20% or more of their body weight. None of the approved thresholds were exceeded at any time. Mice were kept in greenline cages (Tecniplast Typ I superlong) at temperatures between 20 and 24 °C and at 45–60% humidity, receiving pelleted food (1324SP, Altromin) and autoclaved water ad libitum. A light–dark rhythm changed every 12 h with included dimming phases. For peripheral T cell lymphomas, both sexes showed nearly identical trends in survival, although incidence rates were higher in males than in females (https://seer.cancer.gov). In accordance with this, we used both female and male mice. We indicate also that our mouse models did not reveal any differences in PD-1 expression and survival after PD-1 inactivation based upon sex. We used the same numbers of male and female mice in each experiment whenever possible.

### Induction of spontaneous ITK–SYK expression in peripheral T cells

Tamoxifen (T5648, Sigma) was dissolved in pure ethanol (with heating) and subsequently diluted in migliol (3274, Caesar & Loretz) to a final concentration of 10 mg ml^−1^. To induce expression of ITK–SYK in peripheral CD4^+^ T cells, *ITK-SYK*^CD4-CreERT2^ and *ITK-SYK*^CD4-CreERT2^;*Pdcd1*^−/−^ mice were intraperitoneally injected with tamoxifen at the indicated concentrations.

### RNA-seq of mouse samples

*ITK-SYK*^CD4-CreERT2^ or *ITK-SYK*^CD4-CreERT2^;*Pdcd1*^−/−^ mice received a single dose of 2 mg tamoxifen. Five days later, spleens were collected, and 1,000 eGFP^+^ ITK–SYK-expressing CD4^+^ T cells from both genotypes were directly sorted (FACSAria Fusion, BD Biosciences) into a 96-well PCR plate prefilled with 10 µl of 1× TCL buffer (1070498, Qiagen) containing 1% (vol/vol) β-mercaptoethanol (M6250, Sigma). Library preparation for bulk 3′ sequencing of poly(A) RNA was performed as outlined earlier^60^. Briefly, for each sample, barcoded full-length complementary DNA (cDNA) was generated with Maxima RT polymerase (EP0742, Thermo) using oligonucleotide-dT primer-containing barcodes, unique molecular identifiers (UMIs) and an adaptor. The addition of a template switch oligonucleotide resulted in extension of the 5′ ends of the cDNA, and full-length cDNA was amplified with a primer binding to the template switch oligonucleotide site and the adaptor. cDNA was fragmented using the Nextera XT kit (FC-131-1096, Illumina), and only the 3′ end fragments were amplified using primers with Illumina P5 and P7 overhangs. Compared to ref. ^60^, the P5 and P7 sites were exchanged to allow sequencing of the cDNA in read 1 and barcodes and UMIs in read 2 to achieve better cluster recognition. The library was sequenced on the NextSeq 500 platform (Illumina) with 75 cycles for cDNA and 16 cycles for barcodes and UMIs. Raw sequencing data were processed with DropSeq-tools (version 1.12) using gene annotations from the Ensembl GRCm38.87 database to generate sample- and gene-wise UMI tables^61^. Downstream analysis was conducted using R version 3.4.452 and DESeq2 version 1.18.153 (ref. ^62^). Technical replicates with <100,000 total UMIs were excluded before differential expression analysis, and the remaining replicates were collapsed. Genes with <10 reads across all conditions were excluded. Prior differential expression analysis and dispersion of the data were estimated using a parametric fit. GSEA 4.1.0 was used to calculate enrichment for the indicated signatures. All signatures were derived from MSigDB and Harmonizome^51,63–65^.

### Western blot analysis of cytosolic proteins and histones

*ITK-SYK*^CD4-CreERT2^, *ITK-SYK*^CD4-CreERT2^;*Pdcd1*^−/−^ or C57BL/6 mice were intraperitoneally injected with a single dose of 2 mg tamoxifen and received 200 μg anti-PD-L1 antibody (BE0101, BioXcell) or isotype control antibody (BE0090, BioXcell), depending on the experimental condition. At the indicated time points, single-cell suspensions were generated, and eGFP^+^ cells were sorted by flow cytometry and lysed on ice in protein lysis buffer (50 mM Tris, 150 mM NaCl, pH 8, 1% NP-40) supplemented with protease and phosphatase inhibitors (4906845001 and 539131, Sigma). The following antibodies were used at 1:1,000 or 1:10,000 (actin) dilutions for protein detection: anti-hexokinase 2 (ab209847, Abcam), anti-phosphofructokinase 1 (*PFKM*; MAB7687-SP, R&D Systems), anti-aldolase A (8060, CST), anti-enolase 1 (3810T, CST), anti-actin (3700, CST), anti-histone H3 (9715, CST), anti-acetyl-histone H3 Lys27 (8173, CST), anti-histone H4 (2935, CST), anti-acetyl-histone H4 (06-598, Millipore), anti-ACLY (4332, CST), anti-p-ACLY (4331, CST), anti-AKT (4691, CST) and anti-p-AKT (4060, CST).

#### Analysis of p-ACLY^S455^

For the analysis of PD-1-dependent ACLY phosphorylation at serine 455, single-cell suspensions from the spleens of acutely induced *ITK-SYK*^CD4-CreERT2^ mice were incubated overnight with anti-PD-L1 antibody (4 μg ml^−1^, BE0101, BioXcell), isotype control (4 μg ml^−1^, BE0090, BioXcell), DMSO, wortmannin (2 μM, 12-338, Sigma) or LY294002 (10 μM, S1105, SelleckChem). The next day, cells were sorted by flow cytometry for the eGFP^+^ phenotype and lysed as described above.

#### Analysis of histones

For western blotting of histones, single-cell suspensions were generated from the spleens of acutely induced *ITK-SYK*^CD4-CreERT2^ and *ITK-SYK*^CD4-CreERT2^;*Pdcd1*^−/−^ mice. Subsequently, eGFP^+^ cells sorted by flow cytometry were cultured for 4 h in vitro in glucose-free DMEM (11966025, Thermo) supplemented with 1% dialyzed fetal calf serum (FCS; A3382001, Thermo) and 1 mM glucose (G8270, Sigma). Next, cells were washed with ice-cold PBS, and acid-based histone extraction was performed according to published protocols^66^.

### Glucose assay of cell culture supernatants

*ITK-SYK*^CD4-CreERT2^ and *ITK-SYK*^CD4-CreERT2^;*Pdcd1*^−/−^ mice were intraperitoneally injected with a single dose of 2 mg tamoxifen. On day five, single-cell suspensions were generated, and eGFP^+^ cells were sorted by flow cytometry. Two hundred thousand T cells were cultured in vitro in glucose-free DMEM (11966025, Thermo) supplemented with 10% dialyzed FCS (A3382001, Thermo), 2 mM glutamine (25030149, Thermo) and 10 mM glucose for 3 h. The remaining glucose in the supernatants was measured using the Amplex Red Glucose/Glucose Oxidase Assay Kit (A22189, Thermo).

### Seahorse assays

ECAR and OCR were measured using a Seahorse XFe96 Analyzer (Agilent) in XF medium (non-buffered RPMI 1640 medium containing 2 mM glutamine and 1 mM sodium pyruvate (11360070, Thermo) and 25 mM glucose). Two hundred thousand eGFP^+^ ITK–SYK-expressing T cells per well were sorted by flow cytometry and centrifuged onto poly-d-lysine (P6403, Sigma)-coated 96-well plates (101085-004, Agilent) and pre-incubated at 37 °C for 45 min in the absence of CO_2_. Five days before the experiment, *ITK-SYK*^CD4-CreERT2^ and *ITK-SYK*^CD4-CreERT2^;*Pdcd1*^−/−^ mice received a single dose of 2 mg tamoxifen and, depending on the experiment, intraperitoneal injections of anti-PD-L1 antibody (200 μg, BE0101, BioXcell) or isotype control (200 μg, BE0090, BioXcell) as indicated in the figure legends.

### In vivo metabolic imaging using PET–CT and hyperpolarized ^13^C magnetic resonance imaging

C57BL/6, *ITK-SYK*^CD4-CreERT2^ or *ITK-SYK*^CD4-CreERT2^;*Pdcd1*^−/−^ mice received a single dose of 2 mg tamoxifen. For PET–CT imaging, mice were injected with [^18^F]FDG (~12 MBq), and tracer uptake was measured after 90 min using a preclinical Siemens Inveon PET–CT system. Splenic standardized uptake values were calculated using Inveon Research Workplace software. Hyperpolarized ^13^C MRI of the animals was performed using a preclinical 7-T magnetic resonance scanner (Agilent–GE Discovery MR901 magnet and gradient system, Bruker AVANCE III HD electronics). The animals were injected with hyperpolarized [1-^13^C]pyruvate (250 µl, 80 mM, HyperSense DNP, Oxford Instruments), and both [1-^13^C]pyruvate and [1-^13^C]lactate were recorded in vivo using a static 2D FID–CSI (matrix size, 18 × 12; FOV, 30 mm × 20 mm; slice thickness, 3 mm; flip angle, 12°; number of points, 128; bandwidth, 2,000 Hz). *T*_2_-weighted images (^1^H-RARE; matrix size, 150 × 100; FOV, 30 mm × 20 mm; slice thickness, 1 mm) were recorded for co-registration and determination of spleen size. Peak heights for [1-^13^C]pyruvate versus [1-^13^C]lactate were determined and summed over the spleen region of interest using MATLAB code developed in house.

### Intracellular flow cytometry and Phosflow of murine cells

#### Ex vivo

*TK-SYK*^CD4-CreERT2^ or *ITK-SYK*^CD4-CreERT2^;*Pdcd1*^−/−^ mice were intraperitoneally injected with a single dose of 2 mg tamoxifen. At the indicated time points, cells were isolated and immediately fixed in 4% PFA (28906, Thermo), permeabilized using methanol, stained for CD4 (100437, BioLegend), p-AKT^S473^ (48-9715-42, Thermo), p-mTOR^S2448^ (2971, CST), hexokinase 2 (ab209847, Abcam), GLUT1 (PA1-46152, Thermo), p-S6^S240/S244^ (5364S, CST), acetyl-histone H3 Lys27 (13027, CST), HIF1α (36169, CST), Alexa Fluor 647 p-c-FOS^S63^ (8677, CST) and PE p-c-JUN^S73^ (8752, CST) and assessed by flow cytometry. Surface and intracellular antibodies were used at dilutions of 1:300 and 1:100, respectively. The following antibodies were used at a dilution of 1:300 as secondary antibodies: PE donkey anti-rabbit IgG (406421, BioLegend) or PE goat anti-mouse IgG (405307, BioLegend). Data were acquired using a FACSCanto II or an LSRFortessa flow cytometer (BD Biosciences). FlowJo software was used for data analyses (FlowJo).

#### In vitro

eGFP^+^ T cells sorted by flow cytometry from tamoxifen-injected C57BL/6 and *ITK-SYK*^CD4-CreERT2^;*Pdcd1*^−/−^ mice were cultured in vitro in glucose-free DMEM (11966025, Thermo) supplemented with 1% dialyzed FCS (A3382001, Thermo). After 4 h, the cells were fixed in 4% PFA and processed as described above. An exemplified gating strategy is provided in Supplementary Fig. 1.

### Metabolomics

*ITK-SYK*^CD4-CreERT2^ and *ITK-SYK*^CD4-CreERT2^;*Pdcd1*^−/−^ mice were intraperitoneally injected with a single dose of 2 mg tamoxifen per mouse. Five days later, spleen- and lymph node-derived single-cell suspensions were sorted by flow cytometry for the eGFP^+^ phenotype. Subsequently, cells were incubated in glucose-free DMEM (11966025, Thermo) supplemented with 10% dialyzed FCS (A3382001, Thermo) and 11 mM uniformly labeled [U^13^C]glucose (CLM-1396, Cambridge Isotope Laboratories). After 3 h of in vitro culture, cells were washed with ice-cold 0.9% NaCl and resuspended in 1 ml of ice-cold 80% methanol–water solution (vol/vol; 51140, Thermo; AE71.1, Carl Roth), including internal standards, followed by incubation for 8 h at −80 °C. The cell suspension was centrifuged at 13,000*g* and 4 °C for 10 min, and then the supernatants were dried in a SpeedVac concentrator (Savant, Thermo). Lyophilized samples were reconstituted in 40 μl of LC–MS-grade water, vortexed and centrifuged again for 10 min at 13,000*g* and 4 °C. Samples were analyzed using an Agilent 1200 series HPLC system interfaced with an AB Sciex 5500 hybrid triple quadrupole/linear ion trap mass spectrometer equipped with an electrospray ionization source operating in positive or negative mode. Q1 (precursor ion) and Q3 (fragment ion) transitions, the metabolite identifier, dwell times and collision energies for both positive and negative ion modes were used according to published methods with additional transitions for our internal standards^24,67^. Five microliters of sample was injected onto an XBridge Amide HPLC column (3.5 μm; 4.6 mm × 100 mm, 186004868, Waters). Mobile phases were run at 400 μl min^–1^ and consisted of HPLC buffer A (pH 9.0, 95% (vol/vol) water, 5% (vol/vol) acetonitrile (ACN), 20 mM ammonium hydroxide, 20 mM ammonium acetate) and HPLC buffer B (100% ACN). The HPLC settings were as follows: from 0 to 0.1 min, the mobile phase was maintained at 85% buffer B; from 0.1 to 3 min, the percentage of buffer B was decreased from 85% to 30%; from 3 to 12 min, the percentage of buffer B was decreased from 30% to 2% and was maintained at 2% for an additional 3 min. At 15 min, the percentage of buffer B was increased again to 85%, and the column was flushed for an additional 8 min with 85% buffer B. MultiQuant (version 2.1.1, AB Sciex) software was used for data analysis. Metabolite peaks were normalized by cell number and internal standards before statistical analyses.

The retention times for all metabolites were verified using individual purified standards from Sigma: Glycolysis/Gluconeogenesis Metabolite Library (ML0013-1KT), Pentose Phosphate Metabolite Library (ML0012), TCA Cycle Metabolite Library (ML0010)), l-glutathione reduced (G4251), l-glutathione oxidized (G6654), l-serine (S4500) and α-d-glucose 1-phosphate (G6750) using the same chromatographic method. Metabolites were quantified by integrating the chromatographic peak area of the precursor ion.

For measurements without ^13^C labeling, eGFP^+^ cells sorted by flow cytometry from acutely induced *ITK-SYK*^CD4-CreERT2^;*Pdcd1*^−/−^ mice were incubated overnight in vitro in glucose- and glutamine-free DMEM (A1443001, Thermo) supplemented with 10% dialyzed FCS (A3382001, Thermo) and 11 mM glucose (G8270, Sigma), 11 mM galactose (G0750, Sigma) or 11 mM galactose combined with 2 mM *N*-acetylcysteine (A7250, Sigma). The next day, metabolites were isolated and quantified as described above.

For measurement of acetyl-1,2-[^13^C_2_]CoA, cells from the indicated genotypes were prepared as previously described. LC–MS/MS analysis was performed with the following modifications: 10 µl of sample was injected onto an XSelect HSS T3 XP Column (100 Å, 2.5 µm, 2.1 mm × 100 mm, 186006151, Waters). Mobile phases were run at 350 μl min^–1^ and consisted of HPLC buffer A (20 mM ammonium hydroxide, 20 mM ammonium acetate in water (pH 9)) and HPLC buffer B (20 mM ammonium acetate in methanol). The HPLC settings were as follows: from 0 to 1 min, the mobile phase was maintained at 2% buffer B; from 1 to 6 min, the percentage of buffer B was increased from 2% to 80%; from 6 to 9 min, the percentage of buffer B was increased from 80% to 100% and was maintained at 100% for an additional 4 min. At 13 min, the percentage of buffer B was decreased again to 2%, and the column was flushed for an additional 5 min with 2% buffer B. The transitions used are listed in Supplementary Table 1.

To establish the method, the following purified standards were purchased: acetyl-1,2-[^13^C_2_]CoA lithium salt (658650, Sigma), acetyl-CoA sodium salt (A2056, Sigma) and CoA hydrate (C4282, Sigma).

### LC–MS/MS analysis of histone post-translational modifications

*ITK-SYK*^CD4-CreERT2^ and *ITK-SYK*^CD4-CreERT2^;*Pdcd1*^−/−^ mice received a single dose of 2 mg tamoxifen. Five days later, cells were isolated from the spleen, and eGFP^+^ cells were sorted by flow cytometry and cultured in glucose-free DMEM (Thermo, 11966025) supplemented with 10% dialyzed FCS (A3382001, Thermo) and 1 mM [U^13^C]glucose (CLM-1396, Cambridge Isotope Laboratories). After 4 h of in vitro culture, the cells were washed once with PBS and snap frozen in liquid nitrogen. For histone post-translational modification analysis, approximately 1 million cells were extracted with acid. The pelleted cells were resuspended in 100 μl of 0.2 M H_2_SO_4_, and histones were extracted by rotating overnight at 4 °C. Cell debris was removed by centrifugation at 20,817*g* for 10 min at 4 °C. Histones were precipitated by adding trichloroacetic acid (85183, Thermo) to a final concentration of 26%. The tubes were mixed and incubated at 4 °C for 2 h and centrifuged at 20,817*g* for 15 min. Pellets were washed three times with ice-cold 100% acetone (AA22928-K2, VWR) (5 min of rotation at 4 °C, 15 min of centrifugation at 20,817*g* and 4 °C between washes), dried for 15 min at room temperature, resuspended in 20 μl of 1× Laemmli sample buffer per million cells and boiled at 95 °C for 5 min. Samples were stored at −20 °C until further use. Precast polyacrylamide (4–20%) gels (43277.01, Serva) were used to separate the histones corresponding to 0.5 × 10^6^ cells. Gels were briefly stained with InstantBlue Coomassie Protein Stain (ab119211, Abcam). For targeted mass spectrometry analysis, histone bands were excised, washed once with LC–MS-grade water (1153331000, Sigma) and destained twice (or until transparent) by incubating for 30 min at 37 °C with 200 μl of 50% ACN (8825.2, Carl Roth) in 50 mM NH_4_HCO_3_ (T871.1, Carl Roth). Gel pieces were then washed twice with 200 μl LC–MS-grade water and twice with 200 μl of 100% ACN to dehydrate them. Histones were acylated in gel by adding 20 μl of propionic anhydride (175641, Sigma) and 40 μl of 100 mM NH_4_HCO_3_. After 5 min, 140 μl of 1 M NH_4_HCO_3_ was slowly added to the reaction. The pH was confirmed to be approximately 7 for each sample (when the reaction was acidic, a few microliters of 1 M NH_4_HCO_3_ was added). The samples were incubated at 37 °C for 45 min at 550 r.p.m. Afterward, samples were washed five times with 200 μl of 100 mM NH_4_HCO_3_, four times with 200 μl MS-grade water and four times with 200 μl of 100% ACN. They were centrifuged briefly, and all the remaining ACN was removed. Gel pieces were rehydrated in 50 μl trypsin solution (25 ng ml^−1^ trypsin in 100 mM NH_4_HCO_3_) (V5111, Promega) with 1-μl spike tides (SPT-ME-TQL, JPT Peptide Technologies) and incubated at 4 °C for 20 min. After the addition of 150 μl of 50 mM NH_4_HCO_3_, histones were digested in gel overnight at 37 °C and 550 r.p.m. Peptides were sequentially extracted by incubating for 10 min at room temperature with 150 μl of 50 mM NH_4_HCO_3_, twice with 150 μl of 50% ACN (in LC–MS-grade water), 0.1% trifluoroacetic acid (TFA) and twice with 100 μl of 100% ACN. During each of the washing steps, the samples were sonicated for 3 min in a water bath followed by brief centrifugation. The obtained peptides were dried using a centrifugal evaporator and stored at −20 °C until resuspension in 30 μl of 0.1% TFA. For desalting, peptides were loaded in a C18 StageTip (prewashed with 20 μl of methanol followed by 20 μl of 80% ACN and 0.1% TFA and equilibrated with 20 μl of 0.1% TFA), washed twice with 20 μl 0.1% TFA and eluted three times with 10 μl of 80% ACN and 0.25% TFA. The flowthrough obtained from loading peptides in C18 was further desalted with TopTip Carbon (TT1CAR.96, GlyGen) by loading the flowthrough three times (prewashed three times with 30 μl of 100% ACN followed by equilibration three times with 30 μl of 0.1% TFA), washed five times with 30 μl of 0.1% TFA and eluted three times with 15 μl of 70% ACN and 0.1% TFA. Eluted peptides from both desalting steps were combined and evaporated in a centrifugal evaporator, resuspended in 17 μl of 0.1% TFA and stored at −20 °C. The resuspended samples were injected into an UltiMate 3000 RSLCnano system (Thermo) and separated in a 25-cm Aurora column (IonOpticks) with a 50-min gradient from 6% to 43% of 80% ACN in 0.1% formic acid and a 50-min gradient from 5% to 60% ACN in 0.1% formic acid. The effluent from the HPLC was directly electrosprayed into a Q Exactive HF system (Thermo) operated in data-dependent mode to automatically switch between full-scan MS and MS/MS acquisition. Survey full-scan MS spectra (from *m*/*z* 250 to 1,600) were acquired with a resolution of *R* = 60,000 at an *m*/*z* of 400 (AGC target of 3 × 10^6^). The ten most intense peptide ions with charge states between 2 and 5 were sequentially isolated to a target value of 1 × 10^5^ and fragmented at 27% normalized collision energy. Typical mass spectrometric conditions were as follows: spray voltage, 1.5 kV; no sheath and auxiliary gas flow; heated capillary temperature, 250 °C; ion-selection threshold, 33.000 counts. Peak integration was performed using Skyline^68^. Quantified data were further analyzed in R using a previously published formula^69^.

### Chromatin immunoprecipitation followed by sequencing

ChIP–seq was performed as previously described^70^ but with the inclusion of a spike-in strategy to allow for relative quantification of the detected ChIP signal in control versus experimental conditions^71^. Briefly, *ITK-SYK*^CD4-CreERT2^ or *ITK-SYK*^CD4-CreERT2^;*Pdcd1*^−/−^ mice were injected with 2 mg tamoxifen. Five days later, 300,000 cells from the spleens were sorted by flow cytometry for the eGFP^+^ phenotype, immediately cross-linked (1% formaldehyde, 10 min at room temperature), lysed in 100 μl Buffer-B-0.3 (50 mM Tris-HCl, pH 8.0, 10 mM EDTA, 0.3% SDS, 1× protease inhibitors, Roche) and sonicated in a microtube (C300010011, Diagenode) using a Bioruptor Pico device until most of the DNA fragments were 200–500 bp long (settings: temperature, 4 °C; 20 cycles with 30 s on and 30 s off). After shearing and centrifugation at 4 °C and 12,000*g* for 10 min, the supernatant was diluted 1:1 with dilution buffer (1 mM EGTA, 300 mM NaCl, 2% Triton X-100, 0.2% sodium deoxycholate, 1× protease inhibitors, Roche). Sonicated chromatin, supplemented with 50 ng of *Drosophila* spike-in chromatin (53083, Active Motif), was then incubated for 4 h at 4 °C on a rotating wheel with 2 μg anti-H3K27ac (C15410174, Diagenode) and 1 μg anti-H2Av (61686, Active Motif) antibodies conjugated to 15 µl Protein G Dynabeads (10003D, Thermo). Beads were washed five times with buffer A (10 mM Tris-HCl, pH 7.5, 1 mM EDTA, 0.5 mM EGTA, 1% Triton X-100, 0.1% SDS, 0.1% sodium deoxycholate, 140 mM NaCl, 1× protease inhibitors) and once with buffer C (10 mM Tris-HCl, pH 8.0, 10 mM EDTA). Beads were then incubated with 70 μl elution buffer (0.5% SDS, 300 mM NaCl, 5 mM EDTA, 10 mM Tris-HCl, pH 8.0) containing 2 μl proteinase K (20 mg ml^−1^) for 1 h at 55 °C and 8 h at 65 °C to reverse formaldehyde cross-linking, and the supernatant was transferred to a new tube. After adding 30 μl elution buffer to the beads again, the eluates were combined and incubated with another 1 μl of proteinase K for 1 h at 55 °C. Finally, DNA was purified using SPRI AMPure XP beads (A63880, Beckman Coulter) (1:2 sample-to-bead ratio). Purified DNA was used as input for library preparation using the MicroPlex Library Preparation Kit v2 (C05010012, Diagenode) and processed according to the manufacturer’s instructions. Libraries were controlled for quality with the Qubit and Agilent DNA Bioanalyzer. Deep sequencing was performed on HiSeq 1500 systems according to the standard Illumina protocol for 50-bp single-end reads. Reads were aligned to the mouse genome (mm9) and the *Drosophila* genome (dm6) using the Bowtie 2 alignment package^72^. Aligned reads were sorted and indexed using SAMtools (version 1.11)^73^. Sambamba (version 0.8.0) was used to filter out unmapped, multi-mapped and duplicate reads^74^. bamCoverage from deepTools (version 3.3.2) was used with spike-in (*Drosophila*) reads as a normalization factor to extract bigwig files for visualization of data^75^. For differential analysis, peaks were called using the MACS2 package (version 2.2.7.1)^76^. DiffBind (version 2.6.6.2) was used for differential analysis using the built-in spike-in (*Drosophila*) normalization option. GSEA for ChIP–seq peak data was performed using the ChIP-Enrich package (version 2.0.1)^48^.

### Cell culture

Unless otherwise indicated, murine cells were cultured in DMEM containing 20% FCS. The following compounds were used for in vitro experiments: glycolysis inhibitor 2-DG (D8375, Sigma), mTOR inhibitor torin-1 (S2827, SelleckChem), HIF1α inhibitor PX-478 2HCl (S7612, SelleckChem), ACLY inhibitor BMS-303141 (4609, Tocris), fatty acid synthase inhibitor FT113 (S6666, SelleckChem) and BET-p300–CBP dual inhibitor NEO2734 (S9648, SelleckChem). Inhibitors were dissolved in DMSO or water, and the cell culture medium was supplemented with the indicated concentrations of the compound or DMSO or water (as a control).

### Torin-1 inhibitor treatment in vivo

For in vivo treatment with the mTORC1 and mTORC2 inhibitor torin-1, NSG recipient mice received 1 × 10^3^ eGFP^+^ T cells sorted by flow cytometry from *ITK-SYK*^CD4-CreERT2^;*Pdcd1*^−/−^ mice, which had been injected with a single dose of 2 mg tamoxifen (T5648, Sigma) 5 d earlier. Three days after transplantation, NSG mice received torin-1 (S2226, SelleckChem) or vehicle (20% NMP and 50% PEG 400 in ultra-pure water, 494496 and 202398, Sigma) by intraperitoneal injection (10 mg per kg per day), 5 d per week.

### CRISPR–Cas9-mediated deletion of *Acly*

CRISPR–Cas9 ribonucleoproteins (RNPs) were assembled from crRNA–tracrRNA duplexes and Alt-R S.p. Cas9 nuclease (1081059 and 1072534, IDT) according to the manufacturer’s recommendations. Briefly, equimolar amounts of crRNA and tracrRNA were mixed and resuspended in IDTE buffer at a concentration of 44 μM. This mixture was heated to 95 °C for 5 min and allowed to cool to room temperature before adding 36 μM Cas9 enzyme. A resting phase of 20 min at room temperature followed, before the RNPs were used for electroporation. *ITK-SYK*^CD4-CreERT2^;*Pdcd1*^−/−^ mice received a single injection of 2 mg tamoxifen. Five days later, eGFP^+^ cells from the spleen were sorted by flow cytometry. A total of 2 × 10^6^ cells were washed twice with PBS and resuspended in 80 μl P3 solution (V4XP-3032, Lonza), 20 μl RNPs and 1 μl Alt-R Cas9 Electroporation Enhancer (1075916, IDT). Cells were electroporated with a 4D-Nucleofector device (AAF-1002B, Lonza) with pulse code EH115. Immediately after electroporation, 1 ml prewarmed culture medium was added to the cuvettes, and cells were kept at 37 °C for 10 min. Afterward, cells were washed twice, and 1 × 10^6^ eGFP^+^ cells were intravenously injected into wild-type recipient mice with constitutive Cas9 expression (026179, Jackson Laboratory) to avoid the immunogenicity of the Cas9 protein. The following crRNA species were used: GTTCAATGAGAAAGTTCTTG for *Acly* (Mm.Cas9.ACLY.1.AJ) and negative control crRNA 1 (1072544, IDT).

### Isolation of primary human CTCL cells

This study was approved by the Northwestern University Institutional Review Board. Patients provided informed consent for inclusion in the study. Clinical and demographic information on human participants is listed in Supplementary Table 2. Peripheral blood mononuclear cells were isolated from the blood of patients with leukemic CTCL by Ficoll-Hypaque gradient centrifugation (17144003, Cytiva). Leukemic cells were sorted by flow cytometry (FACSAria 5, BD Biosciences) using cell surface markers that uniquely identified the neoplastic clones. If the antibody to TCRvβ was available, we isolated the CD3^+^TCRvβ^+^CD8^−^ population. If not, we isolated CD3^+^CD26^−^CD8^−^ cells. We found that the mutational spectra of cells were similar, regardless of the method of isolation. The resulting CTCL cells had a median purity of >90%. The following antibodies were used: Pacific Blue anti-CD3 (317313, BioLegend), APC anti-CD3 (317318, BioLegend), PerCP-Cy5.5 anti-CD8 (45-0088-42, eBioscience), PE anti-CD26 (302705, BioLegend), PE anti-TCRvβ2 (130-110-095, Miltenyi), FITC anti-TCRvβ13 (11-5792-41, eBioscience), PE anti-TCRvβ14 (130-108-804, Miltenyi) and PE anti-TCRvβ17 (1M2048, Beckman Coulter). For in vitro experiments, CTCL cells and T cells from healthy human donors were cultured in RPMI medium with 10% FCS (Gemini) and 1% penicillin–streptomycin (Gibco). The tumor burden for each patient was determined using well-validated clinical markers, taking whichever value was higher between Sezary cell counts or the CD26-negative cell number by clinical flow cytometry. These were analyzed in conjunction with white blood cell and absolute lymphocyte counts.

### DNA sequencing

Genomic DNA was extracted using a QIAamp Micro Kit (56304, Qiagen). DNA libraries were prepared using a KAPA HyperPrep Kit (KK8504, KAPA Biosystems), and 150-bp paired-end sequencing was performed on an Illumina HiSeq 2000. Sequencing reads were aligned to the human genome (hg19) using the Burrows–Wheeler Aligner^71^.

### Somatic copy number calling

To identify somatic copy number aberrations in whole-genome sequencing data from primary CTCL cells, we used Patchwork (version 2.4) with a window size of 10,000 bp. For quality control, we excluded calls with discordant log_2_ read ratios and delta B allele frequency or high-frequency calls in gnomAD, as previously described^77–79^.

### RNA-seq human and TCR-α and TCR-β sequence analyses

RNA was extracted using the RNeasy Plus Micro Kit (74034, Qiagen). RNA quality was assessed with a Bioanalyzer 2100 (Agilent Technologies), and RNA was quantified using a Qubit RNA HS assay (Q3285, Thermo). cDNA libraries were generated using SMART-Seq version 4 (634891, Clontech) and Nextera XT (FC-131-1024, Illumina) and sequenced on an Illumina HiSeq with a read length of 150-bp paired-end reads. The sequencing reads were aligned using STAR (version 2.4.2), gene-specific transcripts were quantified using HTSeq (version 0.6.0), and differentially expressed transcripts were identified using DESeq2 (version 1.30.1)^62^. MiXCR software was used (version 2.1.10)^80^ to identify TCR sequences from RNA-seq data.

### Phosflow analysis of human cells

Malignant CTCL cells were sorted by flow cytometry as described above. CD4^+^ T cells were immunomagnetically isolated from healthy donor-derived peripheral blood mononuclear cells (11331D, Thermo Scientific). Next, cells were stimulated with anti-CD3 and anti-CD28 beads (1:1 ratio, 11132D, Thermo) for 30 min, washed once with PBS and subsequently fixed and permeabilized (GAS004, Invitrogen). Phosflow staining was performed with anti-p-S6^S240/S244^ (5364S, CST) and secondary Alexa Fluor 647 anti-rabbit IgG Fab2 (4414S, CST) antibodies at dilutions of 1:100. Cells were analyzed using an LSR II flow cytometer (BD Biosciences).

### Experiments with Jurkat T cells

Jurkat cells (clone E6-1, ATCC) and Raji cells (CCL-86, ATCC) were cultured in RPMI with 10% FCS (Gemini) and 1% penicillin–streptomycin (Gibco). For p-c-JUN^S73^ experiments, Jurkat cells transduced with a lentiviral vector encoding *PDCD1* or an empty vector control were co-cultured at a 1:1 ratio with PD-L1-overexpressing Raji cells with or without anti-CD3 and anti-CD28 beads (11161D, Thermo) for 1 h. Fixable live–dead staining was used (L34960, Thermo), and the cells were fixed (554722, BD). Cells were then permeabilized with cold methanol, washed and stained with Alexa Fluor 488-conjugated anti-p-c-JUN^S73^ antibody (12714, CST) at a 1:100 dilution before flow cytometry analysis. For AP-1 reporter assays, cells were transduced with a retrovirus encoding an AP-1 fluorescent reporter construct (Addgene, 118095) with near-infrared fluorescent protein (iRFP) as the fluorescent reporter. AP-1 reporter Jurkat cells were then transduced with *PDCD1* or an empty vector control and co-cultured at a 1:1 ratio with PD-L1 Raji cells with or without anti-CD3 and anti-CD28 beads. After 24 h, AP-1 reporter activity was determined by flow cytometry.

### Glucose-uptake assay in human cells

Malignant CTCL cells were isolated, and CD4^+^ T cells from healthy donors were isolated as described above. Cells were cultured in vitro with anti-CD3 and anti-CD28 beads (1:1 ratio, 11132D, Thermo). The medium was supplemented with the fluorescent glucose analog 2-NBDG (100 μg ml^−1^, 600470, Cayman). At the indicated time points, cells were processed according to the manufacturer’s protocol (600470, Cayman) and analyzed on an LSR II flow cytometer (BD Biosciences).

### Inhibitor treatment of human cells in vitro

Malignant CTCL cells and CD4^+^ T cells from healthy donors were isolated as previously described. Next, cells were stained with the Cell Division Tracker Kit (423801, BioLegend) and cultured in vitro with anti-CD3 and anti-CD28 beads (1:1 ratio, 11132D, Thermo) and 2-DG (S4701, SelleckChem) at the indicated concentrations, everolimus (HY-10218, MedChemExpress), BMS-303141 (SML0784, Sigma) or DMSO vehicle control. After 6 d, the cells were analyzed by flow cytometry, and the division index was determined using the FlowJo proliferation tool (version 10.6, FlowJo).

### Preparation of mouse and human ATAC-seq libraries

#### Ex vivo

For mouse ATAC-seq, *ITK-SYK*^CD4-CreERT2^ or *ITK-SYK*^CD4-CreERT2^;*Pdcd1*^−/−^ mice received a single dose of 2 mg tamoxifen. Five days after the injection, single-cell suspensions were generated from the spleens, and 50,000 eGFP^+^ cells sorted by flow cytometry were used for the transposase reaction. For human ATAC-seq, 50,000 malignant cells from patients with CTCL with and without the *PDCD1* mutation were sorted by flow cytometry as described above. The transposase reaction was performed as previously described^81^. In brief, 50,000 cells were pelleted and resuspended in lysis buffer (10 mM Tris-HCl, pH 7.4, 10 mM NaCl, 3 mM MgCl_2_, 0.1% NP-40 (11332473001, Roche), 0.1% Tween-20 (P1379, Sigma) and 0.01% digitonin (G944A, Sigma)). Lysis was performed on ice for 3 min before washing with 10 mM Tris-HCl, pH 7.4, 10 mM NaCl and 3 mM MgCl_2_ buffer containing 0.1% Tween-20. Nuclei were pelleted, and transposition (Tagment DNA Enzyme I, 15027865, Illumina) was performed at 37 °C for 30 min with shaking at 1,000 r.p.m. in 10 mM Tris-HCl, pH 7.6, 5 mM MgCl_2_, 10% dimethyl formamide (D4551, Sigma), PBS, 0.1% Tween-20, 0.01% digitonin. DNA was column purified (T1030L, NEB), and the transposed DNA was amplified as previously described^81^ in 50-μl reaction volumes with a modified version of custom-designed primers^44^. The reaction was monitored after four cycles with qPCR using a 5-μl PCR reaction mixture and the same primers in a total volume of 15 μl (KK4617, Roche). Cycles were added as calculated, and then the amplified samples were purified and size selected using SpeedBeads (GE65152105050250, Sigma) in 22% PEG. Libraries were quality controlled using the Qubit and Bioanalyzer (5067-4626, Agilent), and 76-bp paired-end sequencing was performed on an Illumina NextSeq 500.

#### In vitro

For ATAC-seq experiments involving inhibitor incubation, single-cell suspensions were generated from spleens of acutely induced *ITK-SYK*^CD4-CreERT2^ or *ITK-SYK*^CD4-CreERT2^;*Pdcd1*^−/−^ mice. Next, cells were incubated in vitro for 3 h in the presence of 5 μM ACLY inhibitor BMS-303141 or DMSO. Finally, ITK–SYK-expressing eGFP^+^ cells were sorted by flow cytometry and immediately processed for ATAC-seq as described above.

### ATAC-seq analysis in mice and humans

For mouse ATAC-seq analysis, Cutadapt (version 3.1) was used to remove adaptor sequences. Next, reads were aligned to the mm9 genome using Bowtie 2 (version 2.4.0)^72^. Afterward, duplicates were marked with MarkDuplicates (Picard, version 2.24.0) and removed with SAMtools (version 1.11). To filter properly mapped reads, we used SAMtools with the following options: exclude multi-mapped reads (MAPQ < 30) and reads with flag 1796 or 1804. Next, SAMtools with the option ‘-f 2’ was used to filter properly paired reads. Adjustment using the Tn*5* shift was performed with alignmentSieve (deepTools, version 3.3.2). Next, we filtered for nucleosome-free reads (0–100 bp) as previously described^44^. Visual inspection after filtering was performed using the bamPEFragmentSize function from deepTools (version 3.3.2). Next, we used MACS2 (version 2.2.7.1) to call ATAC-seq peaks with the following parameters: ‘–nomodel –nolambda –keep-dup auto -call-summits’ (ref. ^76^). HINT-ATAC was used to calculate transcription factor profiles based on JASPAR version 2020 (refs. ^45,46^). chromVAR (version 1.14.0) was used to calculate motif scores^47^. Human ATAC-seq analysis was performed similarly with the following modifications: adaptor sequences were trimmed using Trimmomatic (version 0.36)^82^, and mapping was performed using the hg19 reference genome.

### Statistical analysis of biological experiments and reproducibility

All statistical tests were performed using R software (version 3.5.0 or higher, R Foundation for Statistical Computing). In each experiment, appropriate statistical tests, including non-parametric tests, were used, as indicated in the figure legends. Multiple comparisons were performed using ANOVA, Tukey’s post hoc test and corrected *P* values. Parametric or non-parametric tests were used based on the data distribution. No specific test for normality or equal variances was conducted. No statistical methods were used to predetermine sample size estimates. No data were excluded from the analyses, except one animal that died before the respective time of analysis. The experiments were not randomized, and the investigators were not blinded to allocation during experiments and outcome assessment. The statistical significance level was set at *P* < 0.05.

### Reporting summary

Further information on research design is available in the Nature Portfolio Reporting Summary linked to this article.

### Supplementary information

Supplementary Information Supplementary Fig. 1.

Reporting Summary

Supplementary Tables Supplementary Tables 1–14.

### Source data

Source Data Fig. 1 Statistical source data.

Source Data Fig. 1 Uncropped western blots.

Source Data Fig. 2 Statistical source data.

Source Data Fig. 3 Statistical source data.

Source Data Fig. 3 Uncropped western blots.

Source Data Fig. 4 Statistical source data.

Source Data Fig. 4 Uncropped western blots.

Source Data Fig. 5 Statistical source data.

Source Data Fig. 6 Statistical source data.

Source Data Fig. 7 Statistical source data.

Source Data Extended Data Fig. 1 Statistical source data.

Source Data Extended Data Fig. 2 Statistical source data.

Source Data Extended Data Fig. 3 Statistical source data.

Source Data Extended Data Fig. 4 Statistical source data.

Source Data Extended Data Fig. 6 Statistical source data.

## Data Availability

All data are available from the corresponding authors upon reasonable request. For mouse data, RNA-seq, ATAC-seq and ChIP–seq data have been deposited in the Gene Expression Omnibus database under accessions GSE212832, GSE213180 and GSE183530. For human data, whole-genome sequencing, RNA-seq and ATAC-seq data for consenting patients are deposited in the database of Genotypes and Phenotypes under accession codes phs002456.v1.p1 (for previously published data from ref. ^2^) and phs003312. Raw data from RNA-seq and metabolic experiments can be found in Supplementary Tables 3–14. All other data supporting the findings of this study are available from the corresponding authors on reasonable request. Source data are provided with this paper.

## Code availability

The MATLAB code used to determine the [1-^13^C]pyruvate versus [1-^13^C]lactate peak heights is available from the corresponding authors upon reasonable request.
