# Supplementary material for: PD-1 instructs a tumor-suppressive metabolic program that restricts glycolysis and restrains AP-1 activity in T cell lymphoma
Source: Nat Cancer. 2023 Sep 18;4(10):1508–25. doi: 10.1038/s43018-023-00635-7 (PMC10597841; doi:10.1038/s43018-023-00635-7)
Supplement: Supplementary file 1 — Supplementary Fig. 1. [file 43018_2023_635_MOESM1_ESM.pdf]

nature cancer

Article

https://doi.org/10.1038/s43018-023-00635-7

PD-1 instructs a tumor-suppressive metabolic program that restricts glycolysis and restrains AP-1 activity in T cell lymphoma

In the format provided by the authors and unedited

Exemplified Gating Strategy

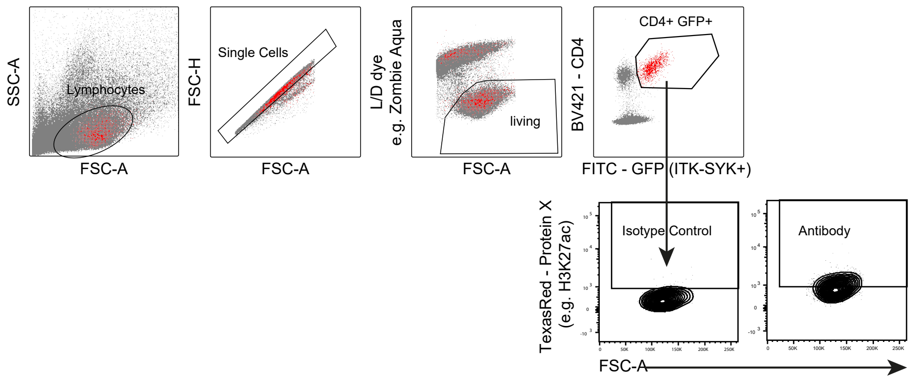

Gating strategy for phosFlow analyses in murine/human tumor cells:
Gate 1: SSC-A and FSC-A were used to determine the lymphocyte gate depending on size
Gate 2: FSC-H and FSC-A were used to exclude duplets
Gate 3: a negative gate is used to exclude deadly-marked cells
Gate 4: CD4+ and FITC+ gate was used to specifically detect tumor cells
Gate 5: proteins of interest; gates have been defined using isotype controls
