# Supplementary material for: PD-1 instructs a tumor-suppressive metabolic program that restricts glycolysis and restrains AP-1 activity in T cell lymphoma
Source: Nat Cancer. 2023 Sep 18;4(10):1508–25. doi: 10.1038/s43018-023-00635-7 (PMC10597841; doi:10.1038/s43018-023-00635-7)
Supplement: Supplementary file 2 — Reporting Summary [file 43018_2023_635_MOESM2_ESM.pdf]

nature portfolio

Corresponding author(s): Prof. Dr. Juergen Ruland

Last updated by author(s): July 31st, 2023

Reporting Summary

Nature Portfolio wishes to improve the reproducibility of the work that we publish. This form provides structure for consistency and transparency in reporting. For further information on Nature Portfolio policies, see our Editorial Policies and the Editorial Policy Checklist.

Statistics

For all statistical analyses, confirm that the following items are present in the figure legend, table legend, main text, or Methods section.

n/a	Confirmed	
☐	☒	The exact sample size (*n*) for each experimental group/condition, given as a discrete number and unit of measurement
☐	☒	A statement on whether measurements were taken from distinct samples or whether the same sample was measured repeatedly
☐	☒	The statistical test(s) used AND whether they are one- or two-sided<br>*Only common tests should be described solely by name; describe more complex techniques in the Methods section.*
☒	☐	A description of all covariates tested
☐	☒	A description of any assumptions or corrections, such as tests of normality and adjustment for multiple comparisons
☐	☒	A full description of the statistical parameters including central tendency (e.g. means) or other basic estimates (e.g. regression coefficient) AND variation (e.g. standard deviation) or associated estimates of uncertainty (e.g. confidence intervals)
☐	☒	For null hypothesis testing, the test statistic (e.g. *F*, *t*, *r*) with confidence intervals, effect sizes, degrees of freedom and *P* value noted<br>*Give P values as exact values whenever suitable.*
☒	☐	For Bayesian analysis, information on the choice of priors and Markov chain Monte Carlo settings
☒	☐	For hierarchical and complex designs, identification of the appropriate level for tests and full reporting of outcomes
☐	☒	Estimates of effect sizes (e.g. Cohen's *d*, Pearson's *r*), indicating how they were calculated

Our web collection on statistics for biologists contains articles on many of the points above.

Software and code

Policy information about availability of computer code

Data collection	BD FACSDiva v9.0.1 was used to collect flow cytometry data.<br>ABSciex Analyst v1.6.3 was used for the ABSciex 5500 hybrid triple quadrupole/linear ion trap mass spectrometer.<br>Thermo XCalibur 4.1.50 was used for the Thermo Qexactive HF hybrid quadrupole-Orbitrap mass spectrometer.<br>HiSeq Control v2.0.12 for Illumina HiSeq 1500 and HiSeq 2000.<br>NextSeq Control v2.2.0 for Illumina NextSeq 500.<br>Agilent Wave v2.3 was used for Agilent Seahorse XFe96 Analyzer.<br>Siemens Inveon Acquistion Workplace v2.0 was used for PET/CT aquisition.<br>Bruker Paravision v6.1 was used for aquisition of hyperpolarized 13C magnetic resonance imaging.
Data analysis	Methods describe the software used to analyze RNA-seq, ATAC-seq, ChIP-seq and Whole-Genome sequencing data in detail and are publicly available.<br>Flowjo v9.3.2 or higher was for flow cytometry.<br>R v3.5.0 or higher was used for statistics.<br>ABSciex MultiQuant v2.1.1. was used for analysis of metabolomics data.<br>Skyline v4.2 was used for analysis of histone PTM peaks.<br>Siemens Inveon Research Workplace v4.2 was used for PET/CT analysis.<br>ImageJ v1.52m for quantification of western blot densities.<br>MathWorks Matlab v.8.5 was used for hyperpolarized 13C magnetic resonance imaging and the used Matlab code is available upon request.<br>MiXCR v2.1.10 was used to identify T cell receptor sequences from RNA-seq data

For manuscripts utilizing custom algorithms or software that are central to the research but not yet described in published literature, software must be made available to editors and reviewers. We strongly encourage code deposition in a community repository (e.g. GitHub). See the Nature Portfolio guidelines for submitting code & software for further information.

Data

Policy information about availability of data

All manuscripts must include a data availability statement. This statement should provide the following information, where applicable:

- Accession codes, unique identifiers, or web links for publicly available datasets
- A description of any restrictions on data availability
- For clinical datasets or third party data, please ensure that the statement adheres to our policy

All data are available from the corresponding authors upon reasonable request. For mouse data, RNA-sequencing, ATAC-sequencing and ChIP-sequencing data have been deposited in the Gene Expression Omnibus database (GEO) with accessions GSE212832, GSE213180, and GSE1835530. For human data, whole genome sequencing, RNA-sequencing and ATAC-sequencing data for consenting patients is deposited in the database of Genotypes and Phenotypes (dbGaP) under the accession codes phs002456.v1 (for previously published data in Park et al, 2021) and phs003312.

Human research participants

Policy information about studies involving human research participants and Sex and Gender in Research.

Reporting on sex and gender	The sex of each patient is reported in Supplemental Table 1. Both male and female patients were analyzed, but sex-specific analyses were not performed due to limitations in the number of patients total.
Population characteristics	Relevant characteristics including ethnicity, sex, age at diagnosis, and the clinical diagnoses are indicated in Supplemental Table 1. This information was determined through patient medical records.
Recruitment	Patients diagnosed with cutaneous T cell lymphoma and who had sufficient stored samples for analysis were included in this study. Participants were recruited through the cutaneous lymphoma clinic at Northwestern University and provided informed consent. This study was approved by the Northwestern University Institutional Review Board.
Ethics oversight	Study protocol was approved by the Northwestern University Institutional Review Board.

Note that full information on the approval of the study protocol must also be provided in the manuscript.

Field-specific reporting

Please select the one below that is the best fit for your research. If you are not sure, read the appropriate sections before making your selection.

☒ Life sciences ☐ Behavioural & social sciences ☐ Ecological, evolutionary & environmental sciences

For a reference copy of the document with all sections, see nature.com/documents/nr-reporting-summary-flat.pdf

Life sciences study design

All studies must disclose on these points even when the disclosure is negative.

Sample size	Samples sizes were not pre-determined based on statistical power calculations but were based on our experience with these assays.
Data exclusions	No data were excluded.
Replication	The number of independent experiments and biological replicates for each data panel is indicated in the figure panels. For human samples, repetition was not always possible due to limited patient material.
Randomization	No randomization techniques were used. However, samples were allocated randomly to experiments and processed in an arbitrary order.
Blinding	The investigators were not blinded to group allocation during data collection or analysis, as there was no subjective measurement in our experiments.

Reporting for specific materials, systems and methods

We require information from authors about some types of materials, experimental systems and methods used in many studies. Here, indicate whether each material, system or method listed is relevant to your study. If you are not sure if a list item applies to your research, read the appropriate section before selecting a response.

Materials & experimental systems

n/a	Involved in the study
☐	☒ Antibodies
☐	☒ Eukaryotic cell lines
☒	☐ Palaeontology and archaeology
☐	☒ Animals and other organisms
☐	☒ Clinical data
☒	☐ Dual use research of concern

Methods

n/a	Involved in the study
☐	☒ ChIP-seq
☐	☒ Flow cytometry
☒	☐ MRI-based neuroimaging

Antibodies

Antibodies used

The following antibodies were used for western blotting (1:1000):
hexokinase 2 (clone epr20839, ab209847, Abcam)
phosphofructokinase-1/PFKM (clone 842735, MAB7687-SP, R&D Systems)
aldolase A (clone D73H4, 8060, CST)
enolase 1 (3810T, CST)
actin (clone 8H10D10, 3700, CST)
histone H3 (9715, CST)
acetyl-histone H3 Lys27 (clone D5E4, 8173, CST)

histone H4 (clone L64C1, 2935, CST)
acetyl-histone H4 (06-598, Millipore)
ACLY (4332, CST)
p-ACLY (4331, CST)
AKT (clone C67E7, 4691, CST)
p-AKT (clone D9E, 4060, CST)

The following antibodies were used for flow cytometry of murine cells:
CD4 (clone GK1.5, 100437, BioLegend); 1:300
p-AKTS473 (clone SDRNR, 48-9715-42, Thermo) ; 1:100
p-mTORS2448 (2971, CST) ; 1:100
hexokinase 2 (clone epr20839, ab209847, Abcam) ; 1:100
glut 1 (21829-1-AP, Proteintech) ; 1:100
p-S6S240/244 (clone D68F8, 5364S, CST) ; 1:100
acetyl-histone H3 Lys27 (clone D5E4, 13027, CST) ; 1:100
HIF1α (clone D1S7W, 36169, CST) ; 1:100
p-c-Jun (9164, CST) ; 1:100
p-c-Fos (clone D82C12, 5348, CST) ; 1:100

The following antibodies were used as secondary antibodies for mouse cells:
PE donkey anti-rabbit IgG (406421, Biolegend) ; 1:300
PE goat anti-mouse IgG (405307, Biolegend) ; 1:300

The following antibodies were used for flow cytometry of human cells:
Pacific blue CD3 (317313, BioLegend) ; 1:300
APC CD3 (clone OKT3, 317318, BioLegend) ; 1:300
PerCPCy5.5 CD8 (clone RPA-T8, 45-0088-42, eBioscience) ; 1:300
PE CD26 (clone BA5b, 302705, Biolegend) ; 1:300
PE TCR Vβ2 (clone REA654, 130-110-095, Miltenyi) ; 1:300
FITC TCR Vβ13 (clone H131, 11-5792-41, eBioscience) ; 1:300
PE TCR Vβ14 (clone REA557, 130-108-804, Miltenyi) , 1:300
PE TCR Vβ17 (clone E17.5F3.15.13, 1M2048, Beckman Coulter) ; 1:300
anti-p-S6S240/244 (clone D68F8, 5364S, CST), AF488 p-c-JunS73 (clone D47G9, 12714, CST); 1:100

The following antibodies were used as secondary antibodies for human cells:
AlexaFluor647 anti-rabbit IgG Fab2 (4414S, CST) ; 1:300

The following antibodies were used for ChIP-seq (amount see methods section):
H3K27ac (C15410174, Diagenode)
H2Av (clone 10E9.D1, 61686, Active Motif)

Validation

All antibodies are commercially available and have been validated by the manufacturer for the applications used in our experiments as indicated on the websites:

Western blotting, mouse:
hexokinase 2 (ab209847, Abcam) https://www.abcam.com/hexokinase-ii-antibody-epr20839-ab209847.html
phosphofructokinase-1/PFKM (MAB7687-SP, R&D Systems) https://www.rndsystems.com/products/human-mouse-rat-muscle-phosphofructokinase-pfkm-antibody-842735_mab7687
aldolase A (8060, CST) https://www.cellsignal.com/products/primary-antibodies/aldolase-a-d73h4-rabbit-mab/8060
enolase 1 (3810T, CST) https://www.cellsignal.com/products/primary-antibodies/enolase-1-antibody/3810

AMPK (5832, CST) https://www.cellsignal.com/products/primary-antibodies/ampka-d63g4-rabbit-mab/5832
tubulin (2144, CST) https://www.cellsignal.com/products/primary-antibodies/a-tubulin-antibody/2144
histone H3 (9715, CST) https://www.cellsignal.com/products/primary-antibodies/histone-h3-antibody/9715
acetyl-histone H3 Lys27 (8173, CST) https://www.cellsignal.com/products/primary-antibodies/acetyl-histone-h3-lys27-d5e4-xp-rabbit-mab/8173
histone H4 (2935, CST) https://www.cellsignal.com/products/primary-antibodies/histone-h4-l64c1-mouse-mab/2935
acetyl-histone H4 (06-598, Millipore) https://www.emdmillipore.com/US/en/product/Anti-acetyl-Histone-H4-Antibody,MM_NF-06-598
ACLY (4332, CST) https://www.cellsignal.com/products/primary-antibodies/atp-citrate-lyase-antibody/4332
p-ACLY (4331, CST) https://www.cellsignal.com/products/primary-antibodies/phospho-atp-citrate-lyase-ser455-antibody/4331
AKT (4691, CST) https://www.cellsignal.com/products/primary-antibodies/akt-pan-c67e7-rabbit-mab/4691
p-AKT (4060, CST) https://www.cellsignal.com/products/primary-antibodies/phospho-akt-ser473-d9e-xp-rabbit-mab/4060

Flow cytometry, mouse:
BV421 CD4 (100437, BioLegend) https://www.biolegend.com/en-us/search-results/brilliant-violet-421-anti-mouse-cd4-antibody-7142
eFluor 450 p-AKTS473 (48-9715-42, Thermo) https://www.thermofisher.com/antibody/product/Phospho-AKT1-Ser473-Antibody-clone-SDRNR-Monoclonal/48-9715-42
p-mTORS2448 (2971, CST) https://www.cellsignal.com/products/primary-antibodies/phospho-mtor-ser2448-antibody/2971
hexokinase 2 (ab209847, Abcam) https://www.abcam.com/hexokinase-ii-antibody-epr20839-ab209847.html
glut 1 (21829-1-AP, Proteintech) https://www.ptglab.com/products/SLC2A1,GLUT1-Antibody-21829-1-AP.htm
p-S6S240/244 (5364S, CST) https://www.cellsignal.com/products/primary-antibodies/phospho-s6-ribosomal-protein-ser240-244-d68f8-xp-rabbit-mab/5364
Alexa Fluor 555 acetyl-histone H3 Lys27 (13027, CST) https://www.cellsignal.com/products/antibody-conjugates/acetyl-histone-h3-lys27-d5e4-xp-rabbit-mab-alexa-fluor-555-conjugate/13027
HIF1α (36169, CST) https://www.cellsignal.com/products/primary-antibodies/hif-1a-d1s7w-xp-rabbit-mab/36169
p-c-Jun (9164, CST) https://www.cellsignal.com/products/primary-antibodies/phospho-c-jun-ser73-antibody/9164

p-c-Fos (5348, CST) https://www.cellsignal.com/products/primary-antibodies/phospho-c-fos-ser32-d82c12-xp-rabbit-mab/5348

PE donkey anti-rabbit IgG (406421, Biolegend) https://www.biolegend.com/en-us/products/pe-donkey-anti-rabbit-igg-minimal-x-reactivity-9751?GroupID=BLG3472
PE goat anti-mouse IgG (405307, Biolegend) https://www.biolegend.com/en-us/products/pe-goat-anti-mouse-igg-minimal-x-reactivity-1418

Flow cytometry, human:
Pacific blue CD3 (317313, BioLegend) https://www.biolegend.com/en-us/products/pacific-blue-anti-human-cd3-antibody-3648?GroupID=BLG4203
APC CD3 (317318, BioLegend) https://www.biolegend.com/en-gb/search-results/apc-anti-human-cd3-antibody-6198
PerCPCy5.5 CD8a (45-0088-42, eBioscience) https://www.thermofisher.com/antibody/product/CD8a-Antibody-clone-RPA-T8-Monoclonal/45-0088-42
PE CD26 (302705, Biolegend) https://www.biolegend.com/en-us/products/pe-anti-human-cd26-antibody-611?GroupID=BLG1985
PE TCR Vβ2 (130-110-095, Miltenyi) https://www.miltenyibiotec.com/US-en/products/tcr-vb2-antibody-anti-human-reafinity-rea654.html#gref
FITC TCR Vβ13 (11-5792-41, eBioscience) https://www.thermofisher.com/antibody/product/TCR-V-beta-13-1-Antibody-clone-H131-Monoclonal/11-5792-41
PE TCR Vβ14 (130-108-804, Miltenyi) https://www.miltenyibiotec.com/US-en/products/tcr-vb14-antibody-anti-human-reafinity-rea557.html#gref
PE TCR Vβ17 (IM2048, Beckman Coulter) https://www.beckman.com/reagents/coulter-flow-cytometry/antibodies-and-kits/single-color-antibodies/tcr-vb17/im2048
anti-p-S6S240/244 (5364S, CST) https://www.cellsignal.com/products/primary-antibodies/phospho-s6-ribosomal-protein-ser240-244-d68f8-xp-rabbit-mab/5364
Alexa Fluor 647 anti-rabbit IgG Fab2 (4414S, CST) https://www.cellsignal.com/products/secondary-antibodies/anti-rabbit-igg-h-l-f-ab-2-fragment-alexa-fluor-647-conjugate/4414

ChIP-seq, mouse:
H3K27ac (C15410174, Diagenode) https://www.diagenode.com/en/p/h3k27ac-polyclonal-antibody-classic-50-mg-42-ml

ChIP-seq, drosophila:
H2Av (61686, Active Motif) https://www.activemotif.com/catalog/details/61751/histone-h2av-antibody-mab-clone-10e9-d1

Eukaryotic cell lines

Policy information about cell lines and Sex and Gender in Research

Cell line source(s)	The Jurkat and Raji cell lines used in this study were purchased from ATCC.
Authentication	All cell lines were authenticated by STR profiling.
Mycoplasma contamination	All cell lines were routinely tested for mycoplasma infection.<br>The tests were always negative.
Commonly misidentified lines<br>(See ICLAC register)	No commonly misidentified cell line was used.

Palaeontology and Archaeology

Specimen provenance	N/A
Specimen deposition	N/A
Dating methods	N/A

☐ Tick this box to confirm that the raw and calibrated dates are available in the paper or in Supplementary Information.

Ethics oversight	N/A

Note that full information on the approval of the study protocol must also be provided in the manuscript.

Animals and other research organisms

Policy information about studies involving animals; ARRIVE guidelines recommended for reporting animal research, and Sex and Gender in Research

Laboratory animals	Mice of both sexes aged 6-12 weeks were used for all experiments. Littermate controls were used whenever possible. Randomization and blinding were not performed. ITK-SYKCD4-creERT2 and ITK-SYKCD4-creERT2;Pdcd1-/- animals were described earlier and maintained on a C57BL/6 genetic background. Pdcd1-/- (02827677), B6J.129(Cg)-Gt(ROSA)26Sortm1.1(CAG-cas9*,-EGFP)Fezh/J (Cas9, 02617978), and NOD.CG-Prkdcscid IL2rgtm1Wjl/SzJ (NSG, 005557) mice were purchased from the Jackson Laboratory.
Wild animals	No wild animals were used.
Reporting on sex	For peripheral T-cell lymphomas both sexes showing nearly identical trends in survival, although incidence rates are higher in males compared to females (seer.cancer.gov). In accordance with this, we used both female and male mice. We indicate also that our mouse models did not reveal any differences in PD-1 expression and survival after PD-1 inactivation based upon sex. We used the same numbers of male and female mice in each experiment whenever possible.
Field-collected samples	No field-collected samples were used.
Ethics oversight	All animal experiments were performed in accordance with local guidelines (Regierung von Oberbayern, Munich, Germany). Mice were euthanized if they exhibited signs of lymphoma (lymph node enlargement, palpable tumor, labored breathing, ascites) or if they lost 20% or more of their body weight. None of the approved thresholds were exceeded at any time.

Note that full information on the approval of the study protocol must also be provided in the manuscript.

Clinical data

Policy information about clinical studies

All manuscripts should comply with the ICMJE guidelines for publication of clinical research and a completed CONSORT checklist must be included with all submissions.

Clinical trial registration	Although this study includes clinical data, this study is not a clinical trial.
Study protocol	Clinical trial study protocols were not utilized. Ethical approval for this study was obtained from institutional review boards.
Data collection	Clinical data was obtained from the electronic medical records of each patient, when available.
Outcomes	We did not predefine outcomes in this study as in a clinical trial.

Dual use research of concern

Policy information about dual use research of concern

Hazards

Could the accidental, deliberate or reckless misuse of agents or technologies generated in the work, or the application of information presented in the manuscript, pose a threat to:

No	Yes	
☒	☐	Public health
☒	☐	National security
☒	☐	Crops and/or livestock
☒	☐	Ecosystems
☒	☐	Any other significant area

Experiments of concern

Does the work involve any of these experiments of concern:

No	Yes	
☒	☐	Demonstrate how to render a vaccine ineffective
☒	☐	Confer resistance to therapeutically useful antibiotics or antiviral agents
☒	☐	Enhance the virulence of a pathogen or render a nonpathogen virulent
☒	☐	Increase transmissibility of a pathogen
☒	☐	Alter the host range of a pathogen
☒	☐	Enable evasion of diagnostic/detection modalities
☒	☐	Enable the weaponization of a biological agent or toxin
☒	☐	Any other potentially harmful combination of experiments and agents

ChIP-seq

Data deposition

☒ Confirm that both raw and final processed data have been deposited in a public database such as GEO.

☒ Confirm that you have deposited or provided access to graph files (e.g. BED files) for the called peaks.

Data access links
May remain private before publication.

https://www.ncbi.nlm.nih.gov/geo/query/acc.cgi?acc=GSE183530
Secure token: qvknycccjdsrxcv

Files in database submission

Raw data files:
GS1296_WT_H3K27ac.r1_ATCACGTT.fastq.gz
GS1301_WT_H3K27ac.r2_GCCAATGT.fastq.gz
GS1297_KO_H3K27ac.r1_CGATGTTT.fastq.gz
GS1302_KO_H3K27ac.r2_CAGATCTG.fastq.gz

Processed data files:
GS1296_WT_H3K27ac.r1_m1_filtered_nodups.ucsc.bigWig
GS1301_WT_H3K27ac.r2_m1_filtered_nodups.ucsc.bigWig
GS1297_KO_H3K27ac.r1_m1_filtered_nodups.ucsc.bigWig
GS1302_KO_H3K27ac.r2_m1_filtered_nodups.ucsc.bigWig

Genome browser session
(e.g. UCSC)

https://genome.ucsc.edu/s/timwartewig/PD1_Metab

Methodology

Replicates

For each genotype, we used two biological replicates

Sequencing depth

Read length: 50bp, single-end

Sample: GS1296_WT_H3K27ac.r1_ATCACGTT
Total Reads: 42284243
Uniquely mapped reads to mm9: 4806047
Uniquely mapped reads to dm6: 977936

Sample: GS1297_KO_H3K27ac.r1_CGATGTTT
Total Reads: 46328389
Uniquely mapped reads to mm9: 38253486
Uniquely mapped reads to dm6: 684970

Sample: GS1301_WT_H3K27ac.r2_GCCAATGT
Total Reads: 47574042
Uniquely mapped reads to mm9: 38620617
Uniquely mapped reads to dm6: 1108937

Sample: GS1302_KO_H3K27ac.r2_CAGATCTG
Total Reads: 49311683
Uniquely mapped reads to mm9: 38934676
Uniquely mapped reads to dm6: 677615

Antibodies

H3K27ac (C15410174, Diagenode), polyclncal, Lot A7071-001P, https://www.diagenode.com/en/p/h3k27ac-polyclonal-antibody-classic-50-mg-42-ml
H2Av (61686, Active Motif), clone 10E9.D1, https://www.activemotif.com/catalog/details/61751/histone-h2av-antibody-mab-clone-10e9-d1

Peak calling parameters

The reads were aligned to the mouse genome (mm9) using the Bowtie2 alignment package:
bowtie2 -p 20 -x ./bowtie2-mm9/bowtie-mm9 -U input.sam

Aligned reads were sorted and indexed using Samtools (v1.11):
samtools view -S -b input.sam > input.bam
samtools sort input.bam -o input.sorted.bam -@ 20

Peaks were called using the MACS2 package (v2.2.7.1) with the calling parameters: macs2 callpeak --broad --broad-cutoff 0.1

Data quality

Spike-in normalisation was used to ensure properly normalised reads after immuno-precipitation and library preparation.
FastQC was used to perform quality control for fastq files. Aligned bam files were visualised via IGV to ensure proper coverage across samples and different loci.
Peaks were called with MACS2 and cross verified.
Peaks above 5-fold enrichment:
GS1296_WT_H3K27ac.r1_ATCACGTT 39365
GS1301_WT_H3K27ac.r2_GCCAATGT 38297
GS1297_KO_H3K27ac.r1_CGATGTTT 39949
GS1302_KO_H3K27ac.r2_CAGATCTG 38888

Software

For differential analysis, the peaks were called using the MACS2 package (v2.2.7.1). DiffBind (v2.6.6.2) was used for differential analysis using the built-in spike-in (Drosophila) normalization option. GSEA for ChIP-seq peak data was performed using the chip-enrich package (v2.0.1).

Flow Cytometry

Plots

Confirm that:

☒ The axis labels state the marker and fluorochrome used (e.g. CD4-FITC).

☒ The axis scales are clearly visible. Include numbers along axes only for bottom left plot of group (a 'group' is an analysis of identical markers).

☒ All plots are contour plots with outliers or pseudocolor plots.

☒ A numerical value for number of cells or percentage (with statistics) is provided.

Methodology

Sample preparation

Mouse sample preparation: Spleens and lymph nodes were gently ground under nylon mesh using the flat end of a 3-mL syringes. Red blood cells were removed by ACK lysing buffer, followed by washing cells with FACS staining buffer (PBS containing 2% FCS). Cells were then filtered, pelleted and stained for FACS or, depending on the experiment, incubated in vitro and subsequently stained for FACS. As described in the methods, fixation and permeabilization was performed prior to staining for the indicated experiments. Collected murine peripheral blood was directly mixed with ACK lysing buffer, followed by washing cells with FACS staining buffer and staining for FACS.
Human sample preparation:
Peripheral blood mononuclear cells (PBMCs) were isolated from the blood of patients with leukemic CTCL by Ficoll-Hypaque gradient centrifugation. Leukemic cells were sorted by FACS using cell surface markers that uniquely identified the neoplastic clones. If the antibody to TCRVβ was available, we isolated the CD3+TCRVβ+CD8− population. If not, we isolated CD3+CD26−CD8− cells. We found that the mutational spectra of cells were similar, regardless of the method of isolation.

Instrument

BD FACSCanto II, BD LSRFortessa, BD LSRII, BD FACSAria Fusion, BD FACSAria 5,

Software

BD FACSDiva 7.0 or higher for sample collection. FlowJo 9.3.2 or higher for FACS analysis.

Cell population abundance

FACS sorted mouse cells had a purity >99%. FACS sorted CTCL cells had a median purity of >90%.

Gating strategy

Based on the pattern of FSC-A/SSC-A, cells in the lymphocyte gate were used for analysis. Singlets were gated according to the pattern of FSC-H vs. FSC-A. Positive populations were determined by the specific antibodies, which were distinct from negative populations. Isotype control was used to distinguish between background and marker-positive events.

☑ Tick this box to confirm that a figure exemplifying the gating strategy is provided in the Supplementary Information.

Magnetic resonance imaging

Experimental design

Design type	N/A
Design specifications	N/A
Behavioral performance measures	N/A

Acquisition

Imaging type(s)	N/A
Field strength	N/A
Sequence & imaging parameters	N/A
Area of acquisition	N/A

Diffusion MRI ☐ Used ☒ Not used

Preprocessing

Preprocessing software	N/A
Normalization	N/A
Normalization template	N/A
Noise and artifact removal	N/A
Volume censoring	N/A

Statistical modeling & inference

Model type and settings	N/A
Effect(s) tested	N/A

Specify type of analysis: ☐ Whole brain ☒ ROI-based ☐ Both

Anatomical location(s)

Statistic type for inference (See Eklund et al. 2016)	N/A
Correction	N/A

Models & analysis

n/a	Involved in the study
☒	☐ Functional and/or effective connectivity
☒	☐ Graph analysis
☒	☐ Multivariate modeling or predictive analysis
