# Supplementary figures and images for: PD-1 instructs a tumor-suppressive metabolic program that restricts glycolysis and restrains AP-1 activity in T cell lymphoma
Source: Nat Cancer. 2023 Sep 18;4(10):1508–25. doi: 10.1038/s43018-023-00635-7 (PMC10597841; doi:10.1038/s43018-023-00635-7)

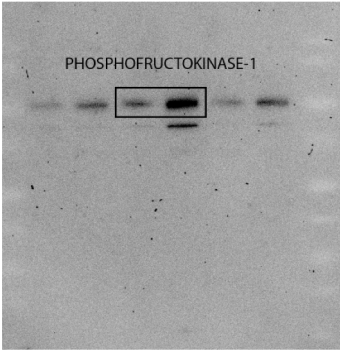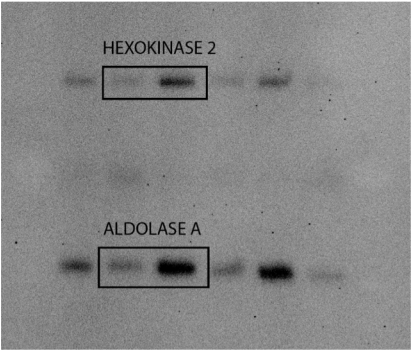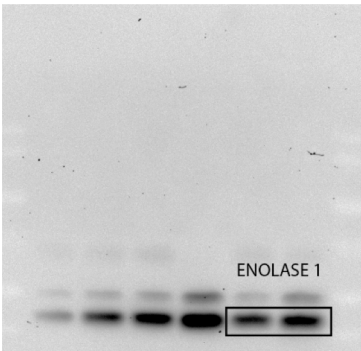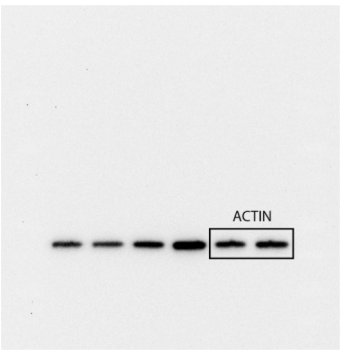

Supplement: Supplementary file 5 — Uncropped western blots. [file 43018_2023_635_MOESM5_ESM.pdf]

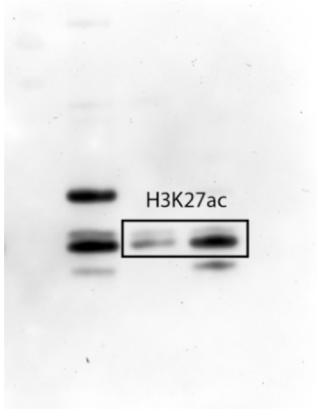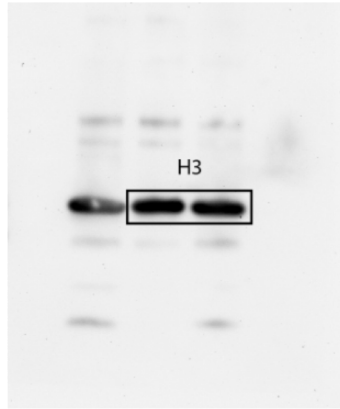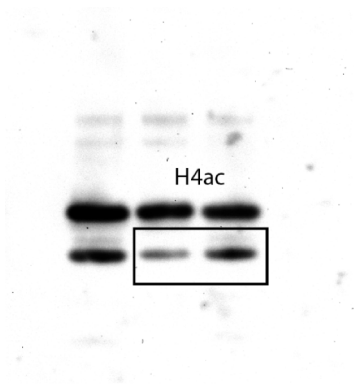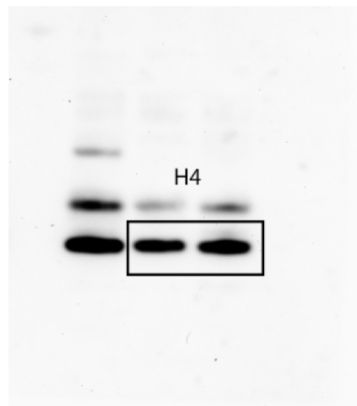

Supplement: Supplementary file 8 — Uncropped western blots. [file 43018_2023_635_MOESM8_ESM.pdf]

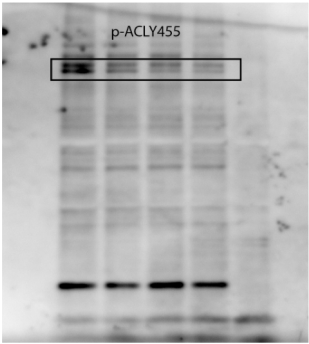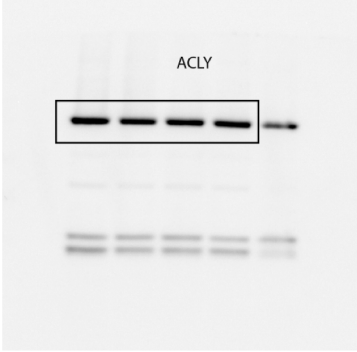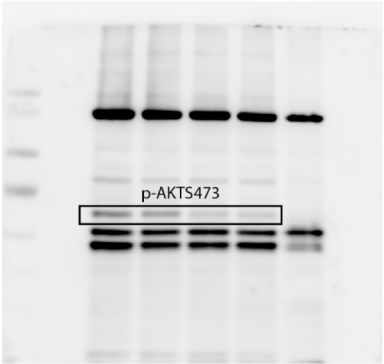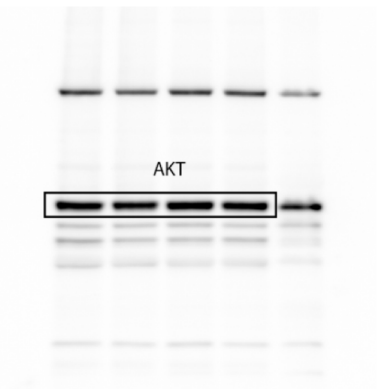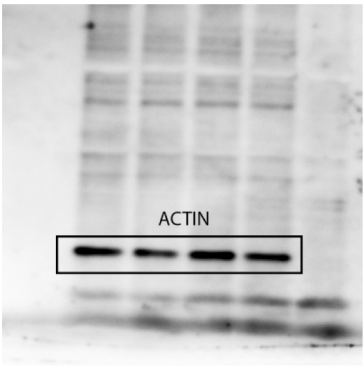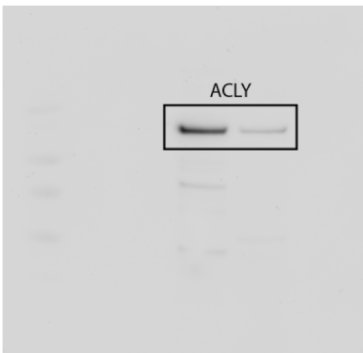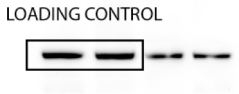

Supplement: Supplementary file 10 — Uncropped western blots. [file 43018_2023_635_MOESM10_ESM.pdf]
